# Helicobacter pylori infection and lactose intolerance increase expiratory hydrogen

**DOI:** 10.17179/excli2021-4508

**Published:** 2022-02-17

**Authors:** Wolfgang J. Schnedl, Nathalie Meier-Allard, Michael Schenk, Sonja Lackner, Dietmar Enko, Harald Mangge, Sandra J. Holasek

**Affiliations:** 1 Practice for General Internal Medicine, Dr. Theodor Körnerstrasse 19b, A-8600 Bruck/Mur, Austria; 2 Division of Immunology and Pathophysiology, Otto Loewi Research Center, Medical University of Graz, Heinrichstrasse 31a, A-8010 Graz, Austria; 3 Das Kinderwunsch Institut Schenk GmbH, Am Sendergrund 11, A-8143 Dobl, Austria; 4 Clinical Institute of Medical and Chemical Laboratory Diagnosis, Medical University of Graz, Auenbruggerplatz 30, A-8036 Graz, Austria

**Keywords:** irritable bowel syndrome, lactose intolerance, histamine intolerance, diamine oxidase, fructose malabsorption

## Abstract

Infection with *Helicobacter pylori* (*H.pylori*) may cause dyspepsia and/or unexplained functional nonspecific, gastrointestinal complaints of the irritable bowel syndrome (IBS) spectrum. Hitherto, in *H. pylori* infected patients with symptoms of the IBS spectrum the occurrence of additional food intolerance/malabsorption is not evaluated. We used a retrospective analysis of charts from 548 patients who presented with gastrointestinal complaints of the irritable bowel syndrome spectrum. An enzyme-linked IgA immunosorbent assay or histologic evaluation of gastric mucosa were used to detect *H. pylori *infection. A hydrogen breath (H_2_) test was performed to evaluate fructose malabsorption (FM) and lactose intolerance (LIT). Serum diamine oxidase value of <10 U/ml and a response to a histamine-reduced diet was used to identify histamine intolerance (HIT). We found 293 patients infected with* H. pylori*, within these were 58 *H. pylori *patients with LIT, 23 *H. pylori *LIT patients with FM and 46 *H. pylori *LIT patients with HIT. Additionally, 13 *H. pylori,* lactose- and histamine intolerance patients also had FM. The Kruskal Wallis test and pairwise comparison were used to analyze differences of the area under the curve of expiratory hydrogen. In lactose H_2_ breath tests compared with LIT-only patients, LIT with *H. pylori*, LIT and *H. pylori* with HIT, LIT and *H. pylori* with FM showed significantly higher exhaled H_2_ levels (p=0.022). Pairwise comparison demonstrated *H. pylori* infected patients with LIT exhaled more H_2_ compared to LIT-only (p=0.029). *H. pylori *with lactose- and histamine intolerance, and *H. pylori *with lactose-, histamine intolerance and FM compared to *H. pylori*-only patients indicated a significantly higher occurrence of stomach pain during lactose H_2_ breath tests (p=0.012 and p=0.005, respectively). We demonstrate that LIT patients with high expiratory H_2_ levels in lactose breath tests may have *H. pylori* infection and possibly additional food intolerance/malabsorption. Subsequently, besides *H. pylori *eradication, a dietician is necessary for an individually tailored reduction- or exclusion diet of symptom triggering food components.

## Abbreviations

AUC, Area under the curve; DAO, diamine oxidase; FM, fructose malabsorption; GI, gastrointestinal; HIT, histamine intolerance; IBS, irritable bowel syndrome; LIT, lactose intolerance.

## Introduction

*Helicobacter pylori* (*H. pylori*) infection is the most prevalent human pathogen and is present in more than 50 % of worldwide populations. If *H. pylori* is detected, then an eradication therapy is mandatory. Some association of *H. pylori* infection and dyspepsia and/or unexplained functional, nonspecific, non-allergic gastrointestinal (GI) complaints of the irritable bowel syndrome (IBS) spectrum is documented. Reduction of these symptoms due to *H. pylori* eradication has been shown (Schulz and Kupčinskas, 2020[22]). Based on current recommendations, patients with *H. pylori*-associated dyspepsia/unexplained functional, nonspecific, non-allergic GI complaints may be considered as a separate group of patients with functional dyspepsia (McNicholl et al., 2019[13]).

Unexplained functional, nonspecific, non-allergic GI complaints and dyspepsia, including IBS and IBS-like syndromes, are widespread and costly, and a main reason for consultations in primary care settings (Camilleri, 2021[5]). Generally, pathophysiology of symptoms within the IBS spectrum is not entirely understood. Nonetheless, these symptom-based syndromes have a symptom profile comparable to that of food intolerance/malabsorption (Lacy et al., 2021[12]). Food intolerance/malabsorption includes celiac disease (CD), fructose malabsorption (FM), lactose intolerance (LIT) (Harer and Eswaran, 2021[9]) and histamine intolerance (HIT). Abdominal complaints caused by LIT and FM appear when these sugars are not appropriately degraded and digested (Storhaug et al., 2017[24]; Basu et al., 2021[1]). In HIT a disproportionate amount of histamine in the intestine is supposed to result from the consumption of food with high histamine content. Apparently there exists a reduced ability of the enzyme diamine oxidase (DAO) to degrade histamine (Comas-Basté et al., 2020[6]). 

In this study we have evaluated *H. pylori*-infected patients for additional food intolerance/malabsorption, including CD, FM, HIT and LIT. All included patients presented with dyspepsia/unexplained functional, nonspecific, non-allergic GI complaints of the IBS spectrum. Compared to LIT-only, patients with LIT and *H. pylori *combined with or without additional food intolerance/malabsorption demonstrated significantly higher expiratory hydrogen (H_2_) values during lactose tolerance breath tests. 

## Methods

During the retrospective evaluation, we found 548 patients who presented with dyspepsia/unexplained functional, nonspecific, non-allergic GI complaints of the IBS spectrum. Main symptoms included abdominal pain, diarrhea, loose stools, bloating, constipation and postprandial fullness. They were examined for additional food intolerance/malabsorption, including CD, FM, HIT, and LIT. In evaluated patients, either an enzyme-linked IgA immunosorbent assay (ELISA, Serion, Würzburg, Germany) or a histologic evaluation of gastric mucosa was used to detect *H. pylori* infection. 293 were infected with* H. pylori* and in 255 *H. pylori* was not found*.* Eradication therapy was started after performance of H_2_ breath tests and included in this evaluation were only patients who took part in all tests. Patients with alarming symptoms, such as vomiting, rectal bleeding or unintentional weight loss were excluded.

H_2_ breath tests (Gastrolyzer, Bedfont Scientific Inc., Kent, England) were used for LIT and FM testing as described earlier (Schnedl et al., 2020[19]). After overnight fasting (>12 hours) blood drawings were performed in the morning, and an H_2_ breath testing was started. In our outpatient setting, we include determination of DAO values in the evaluation of patients presenting with dyspepsia/unexplained functional, nonspecific, non-allergic GI complaints of the IBS spectrum. The DAO values were determined through the radio extraction assay, DAO Rea 100 (Sciotec Diagnostic Technologies, Tulln, Austria).

A thorough anamnesis, concerning abdominal complaints, and a timely relation to the ingestion of food or drinks, including pharmaceutical treatments that might influence dyspepsia/unexplained functional, nonspecific, non-allergic GI complaints of the IBS spectrum was performed. A registered dietitian was consulted to implement and monitor an individualized diet to reduce and/or eliminate symptom-triggering foods. For screening of celiac disease, antibodies against tissue transglutaminase were measured with the anti-tTG IgA ELISA (Euro Diagnostica AB, Malmö, Sweden) method. 

The study follows the ethical guidelines of the Declaration of Helsinki and was approved by the Ethical Committee of the Johannes Kepler University in Linz, Austria (No. K-107-16).

## Statistical Analysis

Descriptive statistics are presented as medians with interquartile ranges (IQR). The data distribution was assessed with a Shapiro Wilk test. For not normally distributed data non-parametric tests were applied. The areas under the curve (AUC) of exhaled hydrogen during H_2_ breath tests were calculated and compared with the Kruskal-Wallis test, and pairwise comparison. The Chi-square and the Fisher's exact test were applied for evaluation of symptoms. The level of significance was set to 5 %. The Bonferroni correction was used for multiple testing.

Statistical analyses were performed with IBM SPSS statistics version 25.0 (IBM, Armonk, NY, USA), and GraphPad Prism version 9.0.0. (GraphPad Software, San Diego, CA, USA) was used for the generation of figures.

## Results

For this retrospective evaluation, we used 548 patients with IBS spectrum symptoms. We found 293 infected with* H. pylori*, male/female 93/200, median age 50 years (IQR 21), age range 17-93 years. We identified 68 patients with *H. pylori *infection only (12.4 %) - male/female 25/43, median age 56 years (IQR 22), age range 25-93 years. Hydrogen breath tests neither showed LIT nor FM in these patients, and serum DAO values were within normal >10 IU/mL (median 17.9 IU/mL (IQR 12.1), range 10.1-63.4 IU/mL). 58 patients with *H. pylori* had additional LIT (10.6 %) - male/female 20/38, median age 50 years (IQR 17), age range 22-84 years. Hydrogen breath tests did not show FM in these patients, and DAO values were >10 IU/mL (median 17 IU/mL (IQR 9.5), range 10.5-80 IU/mL). 17 *H. pylori* patients had only additional FM (3.1 %), male/female 3/14, median age 57 years (IQR 15), age range 23-78 years. Hydrogen breath tests did not show LIT in these patients, and DAO values were >10 IU/mL (median 14.7 IU/mL (IQR 5.6), range 10.7-30.6 IU/mL). However, 47 *H. pylori* patients had additional HIT (8.6 %), male/female 12/35, median age 50 years (IQR 22), age range 24-91 years. Their DAO values were <10 IU/mL (median 5 IU/mL (IQR 5.2), range 1.5-9.7 IU/mL). Moreover, hydrogen breath tests did show neither LIT nor FM in this group. 

Additionally, 23 patients showed *H. pylori* combined with LIT and FM (4.2 %) - male/female 6/17, median age 45 years (IQR 21), and age range 17-82 years. Their DAO values were >10 IU/mL (median 16.6 IU/mL (IQR 9.5), range 12.4-71 IU/mL). In 46 patients we found *H. pylori* combined with LIT and HIT (8.4 %) - male/female 17/29, median age 43 years (IQR 21), age range 22-83 years. Accordingly, their DAO values were <10 IU/mL (median 6.6 IU/mL (IQR 3.0), range 1.5-9.9 IU/mL) and hydrogen breath tests did not show FM. In 17 *H. pylori* patients we found additional FM combined with HIT (3.1 %) - male/female 6/11, median age 54 years (IQR 22), age range 28-79 years. Their DAO values were <10 IU/mL (median 4.1 IU/mL (IQR 6.7), range 1.5-10 IU/mL). Hydrogen breath tests did not show LIT in these patients. 13 patients with *H. pylori* had LIT and FM combined with HIT (2.4 %). Their DAO values were <10 IU/mL (median 7.8 IU/mL (IQR 3.5), range 2.1-9.9 IU/mL). Antibodies against tissue transglutaminase were not detectable in 289 *H. pylori* patients. Four patients with *H. pylori* infection demonstrated celiac disease (0.8 %) with elevated tissue transglutaminase antibodies and following histologic diagnosis of the duodenal mucosa as shown in Table 1(Tab. 1).

In 153 (27.9 %) patients (male/female 64/89 median age 41 (IQR 24), age range 16-84 years, LIT only was diagnosed. Their DAO values were >10 IU/mL (median 16.6U/ml (IQR 10.2), range 10.2-80 IU/mL) and they did not show FM in fructose H_2_ breath tests. 102 patients (18.6 %) were positive for FM (male/female 34/68, median age 46 years (IQR 29), range 19-91 years). Their DAO values were > 10 IU/mL, with median 16.9 IU/mL (IQR 7.9) and range 10.2-62.5 IU/mL and they did not show LIT in lactose H_2_ breath tests. In all of these 255 patients neither antibodies against *H. pylori* nor tissue transglutaminase were detected.

**Figure 1:**
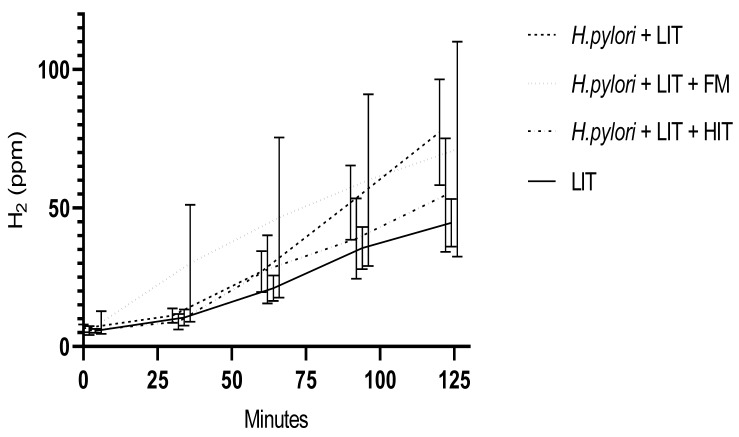
Medians and 95 % confidence intervals of rising H_2_ in parts per million (ppm) during lactose breath tests in 433 LIT patients with >20 ppm increase of expiratory H_2_ from baseline. Included are 153 LIT-only patients without *H. pylori *infection. Of 280 *H. pylori *infected LIT patients 58 were with additional LIT only, 46 *H. pylori* patients with LIT and HIT, and 23 *H. pylori* patients with LIT and FM. Abbreviations: FM, Fructose malabsorption; *H.pylori*, *Helicobacter pylori;* HIT, histamine intolerance; H_2_, hydrogen; LIT, lactose intolerance; ppm, parts per million

The Kruskal Wallis test for the comparison of the AUCs of LIT-only, to patients with LIT and *H. pylori*, to *H. pylori*, LIT with HIT and, to *H. pylori*, LIT with FM demonstrated significantly higher exhaled hydrogen values (p=0.022) (Figure 1(Fig. 1)). Pairwise comparison demonstrated *H. pylori* infected patients with LIT exhaled more H_2_ compared to LIT-only (p=0,029), corrected for multiple testing (test statistic 35.206 and standard error 12.48). Due to the low number of *H. pylori* patients with LIT, FM and HIT these were not included in Figure 1(Fig. 1). However, if included, we calculated p=0.054.

**Figure 2:**
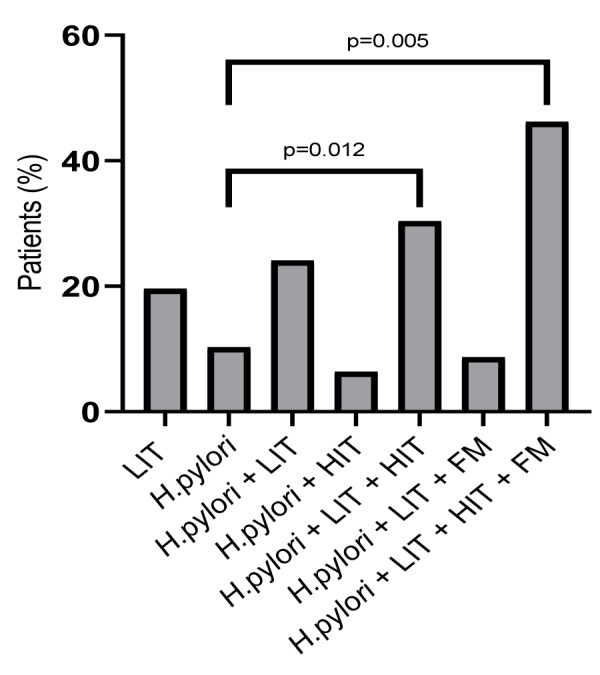
Sixty nine of 378 LIT patients indicated abdominal pain during lactose H_2_ breath tests. Abdominal pain was specified by 30 of 153 LIT patients (19.6 %), seven of 68 patients with *H. pylori* (10.3 %), 14 patients of 58 *H. pylori* and LIT patients (24.1 %), three of 47 *H. pylori* and HIT patients (6.4 %), 14 of 46* H. pylori* and LIT with HIT patients (33.4 %), two of 23 *H. pylori*, LIT and FM patients (8.7 %) and in six patients of 13 *H. pylori*, LIT, HIT with FM patients (46.2 %). Abbreviations: FM, Fructose malabsorption; *H. pylori*, *Helicobacter pylori;* HIT, histamine intolerance; LIT, lactose intolerance

During performed lactose breath tests patients specified their GI and extra-intestinal symptoms on paper. Indicated GI symptoms by *H. pylori *and LIT patients were abdominal pain, bloating, diarrhea, and increased bowel movements. Other GI symptoms included nausea, heartburn, belching, fullness, and excessive mucus in the throat. Extra-intestinal symptoms were headache, fatigue, vertigo and one eczema. Various double and few triple combinations of these symptoms were indicated. GI-symptoms were indicated by 160 of 279 LIT patients (57 %) during H_2_ breath tests. As shown in Figure 2(Fig. 2), *H. pylori* with LIT and FM, and *H. pylori* with LIT, FM and HIT compared to *H. pylori*-only patients demonstrate significantly higher occurrence of stomach pain (p=0.012 and p=0,005, respectively). Patients with *H. pylori-*only infection did specify symptoms, although neither bloating nor diarrhea were mentioned during lactose breath tests. All patients >50 years old were screened by colonoscopy and no pathology was present.

The enzyme-linked IgA immunosorbent assay (ELISA, Serion, Würzburg, Germany) detected IgA antibodies against *H. pylori* in 262 patients, male/female 84/178, median age 51 years (IQR 22), age range 17-93 years, with mean 77.2 IU/mL (range 21-200 IU/mL) IgA antibodies. 63 patients had *H. pylori *only, male/female 25/38, median age 53 years (IQR 24), age range 25-93 years, and they had mean 73.9 IU/mL (range 21-200 IU/mL) IgA antibodies. 45 patients had *H. pylori* combined with LIT, male/female 17/28, median age 49 years (IQR 16), age range 26-86 years, with mean 63.8 IU/mL (range 21-200 IU/mL) IgA antibodies. 46 were *H. pylori* and HIT patients male/female 9/37, median age 49.5 years (IQR 24), age range 24-91 years, they had IgA antibodies mean 65.3 IU/mL (range 22-200 IU/mL). 19 were *H. pylori* and FM patients, male/female 3/16, and median age 56 years (IQR 17), age range 23-78 years, with mean 74.1 IU/mL (range 21-200 IU/mL) IgA antibodies. 44 were *H. pylori* and LIT with HIT patients, male/female 17/27, median age 43 years (IQR 18), age range 22-83 years, IgA antibodies with mean 84.9 IU/mL (range 21-200 IU/mL). 19 were *H. pylori* patients with LIT and FM male/female 6/13, median age 51 years (IQR 21), age range 17-79 years, with mean 111 IU/mL (range 34-200 IU/mL) IgA antibodies. 15 were *H. pylori*, FM and HIT patients male/female 5/10, median age 54 years (IQR 28), age range 28-58 years, IgA antibodies with mean 95.3 IU/mL (range 24-200 IU/mL). 11 *H. pylori*, LIT, HIT with FM patients, male/female 2/9, median age 41 years (IQR 32), age range 24-83 years, with mean 92.8 IU/mL (range 25-200 IU/mL) IgA antibodies.

**Figure 3:**
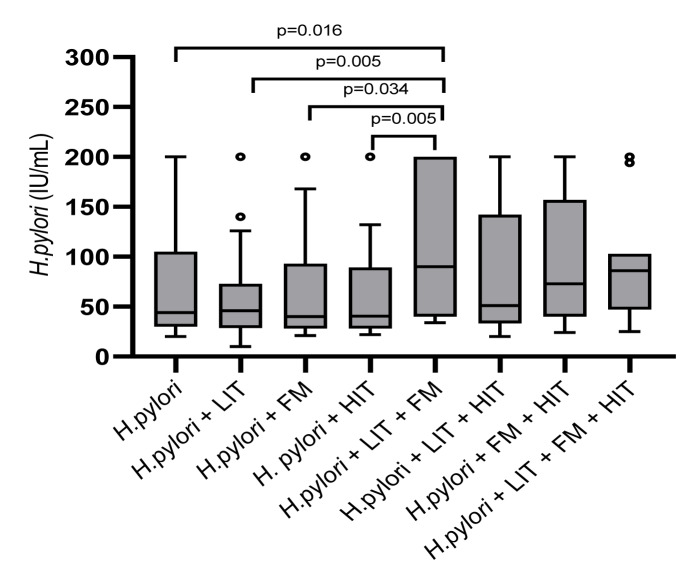
In 262 patients IgA antibodies against *H. pylori* were detected with enzyme-linked IgA immunosorbent assay*.* Included are 63 patients with *H. pylori *only, 45 patients *H. pylori* combined with LIT, 19 *H. pylori* and FM patients, 46 *H. pylori* with HIT, 19 were *H. pylori* patients with LIT and FM, 44 were *H. pylori* and LIT with HIT patients, 15 *H. pylori*, FM and HIT and 11 *H. pylori*, LIT, HIT with FM patients. Abbreviations: FM, Fructose malabsorption; *H. pylori*, *Helicobacter pylori;* HIT, histamine intolerance; LIT, lactose intolerance

As shown in Figure 3(Fig. 3), the Kruskal-Wallis test showed antibody values against *H. pylori* significant different among all groups (p=0.044). Patients with *H. pylori*, LIT and FM had the highest quantity of IgA antibodies. Pairwise comparisons showed significantly lower antibody values comparing *H. pylori*, LIT and FM to *H. pylori* and LIT (p=0.005), to *H. pylori* and HIT (p=0.005), to *H. pylori* with FM (p=0.034) and, to *H. pylori* only (p=0.016). However, correction for multiple testing showed no significance.

Fructose malabsorption breath tests (n=159), using the Kruskall-Wallis test for evaluation of AUCs, showed no differences of exhaled H_2_ (p=0.39) comparing all groups (data not shown). 

See also the Supplementary data.

## Discussion

From a clinical perspective, considerable overlap exists between food intolerance/ malabsorption and unexplained dyspepsia/ functional, nonspecific, non-allergic GI complaints and disorders, including IBS and IBS-like disorders. Currently, IBS is classified as a functional gastrointestinal disorder, according to Rome IV criteria, but new disease models are being proposed continuously (Talley, 2020[26]). Food intolerance/malabsorption syndromes may cause dyspepsia/unexplained functional, nonspecific, non-allergic GI complaints and extra-intestinal symptoms appearing in up to 20 % of populations. Chronic unexplained GI complaints, as caused by IBS and IBS-like syndromes, are only symptom-based. They are associated with high symptom burden and a reduced quality of life. Generally, lactose is a known main trigger causing dyspepsia/ functional, nonspecific, non-allergic GI complaints within the IBS spectrum (Storhaug et al., 2017[24]). FM and LIT are frequent but underestimated conditions in patients with IBS-like symptoms (Wilder-Smith et al., 2020[28]). However, IBS patients claim that food, including histamine-containing food, may cause their GI symptoms (Böhn et al., 2013[2]). Dietary adaptations, targeting exclusion or limited consumption of symptom-triggering food, after an individual diagnostic evaluation of food intolerance/ malabsorption, demonstrate clinical benefits (Schnedl et al., 2020[20]).

Combined with *H. pylori, *the hormone gastrin and the biogenic amine histamine are main stimulants of gastric acid secretion (Schubert, 2017[21]). In patients with unexplained dyspepsia/functional, nonspecific, non-allergic GI complaints of the IBS spectrum, an *H. pylori* infection should be considered, too. Accordingly, if it is detected, an eradication therapy is required (Tomita et al., 2018[27]). Due to symptoms of the IBS spectrum being most common in women and young people without alarming symptoms (Camilleri, 2021[5]), we used serology as a non-invasive test for the initial diagnosis of *H. pylori*. Support for the use of this noninvasive diagnostic method in the initial detection of *H. pylori* gastritis has been published (Bosch et al., 2020[3]).

*H. pylori* infection and its association with deficiencies of micronutrients, minerals and vitamins was reported (Franceschi et al., 2014[8]). A relationship between *H. pylori* and iron deficiency has been established and recommended treatment can be found in the Maastricht IV European guidelines (Talley, 2020[26]). Successful *H. pylori *eradication was shown to improve iron and vitamin B12 absorption (Kaptan et al., 2000[10]). Additionally, a relationship between *H. pylori* infection and impaired absorption of certain orally administered medications and their reduced bioavailability has been suggested (Stillhart et al., 2020[23]). Several guidelines and consensus reports have been published to manage dyspepsia/functional, nonspecific, non-allergic GI complaints and *H. pylori *infection (Schulz and Kupčinskas, 2020[22]). Finally, the influences of additional food intolerance/malabsorption, including CD, FM, and HIT in *H. pylori-*infected LIT patients with dyspepsia/functional, nonspecific, non-allergic GI complaints within the IBS spectrum, may add new information. 

Overall, endoscopy with biopsies and histological evaluation of GI mucosa and radiology, including ultrasound, are not exchangeable methods for the exclusion of other organic diseases, especially in patients with complaints of the IBS spectrum aged >50 years. Nonetheless, for an evaluation of *H. pylori *patients with dyspepsia/functional, nonspecific, non-allergic GI complaints of the IBS spectrum, besides a thorough anamnesis, it seems useful to include examinations for food intolerance/malabsorption with FM- and lactose intolerance H_2_ breath tests, serum DAO determination and screening for celiac disease (Schnedl and Enko, 2021[17]). 

Symptoms in LIT and FM may appear, when the sugars, lactose and fructose, pass through the intestines without being degraded and/or absorbed properly. In intolerance/ malabsorption, they reach the microbiota, where they act as bacterial substrate. Although, the impact of the gut microbiome on the occurrence of gut-related symptoms, especially in LIT, remains unclear (Brandao Gois et al., 2022[4]), fermentation results in H_2_ production. Therefore, the clinical diagnosis of LIT and FM is primarily performed with H_2_ breath tests. Impaired degradation of ingested histamine due to an anticipated gastrointestinal DAO deficiency, promotes HIT. So far, serum DAO is not established to reflect GI histamine-degrading DAO activity (Reese et al. 2021[16]). Nonetheless, increasing data on the association between serum DAO and GI histamine-degrading DAO activity are being demonstrated. Moreover, the diagnosis of HIT, in patients with dyspepsia/functional, nonspecific, non-allergic GI complaints of the IBS spectrum may be supported with serum DAO measurements (Lackner et al. 2019[11]; Mušič et al., 2013[14]). Although, it was suggested that HIT originates in the gut, further studies on the challenging recognition and an improvement of diagnostic methods are necessary (Schnedl and Enko, 2021[18]).

Generally, only few studies have reported on combined occurrence of food intolerance/malabsorption. Nonetheless, more than 30 % of patients with dyspepsia/ unexplained functional, nonspecific, non-allergic GI complaints and carbohydrate intolerance/malabsorption may have combinations of these (Wilder-Smith et al., 2020[28]). Next to the use of a standardized questionnaire for anamnesis of symptoms we decided, to include determination of serum DAO, as a low DAO may help to indicate HIT, in evaluation of patients with dyspepsia/functional, nonspecific, non-allergic GI complaints of the IBS spectrum (Schnedl and Enko, 2021[18]). A parallel occurrence of low serum DAO values <10 U/mL was described in more than 50 % of patients with LIT and FM (Enko et al., 2016[7]). So far, there are very few studies exploring HIT in patients with dyspepsia/functional, nonspecific, non-allergic GI complaints of the IBS spectrum. We present a single center experience, suggesting that combinations of food intolerance/malabsorption, including HIT, offer a diagnosis which may help patients to put dyspepsia/unexplained functional, nonspecific, non-allergic GI complaints of the IBS spectrum into context. 

In patients with LIT, without *H. pylori*, the presence of additional food intolerance/ malabsorption induced significantly increased expiratory H_2_ values (Schnedl et al., 2020[19]). In this study we demonstrate that in patients with LIT, the additional presence of *H. pylori* infection, with or without further food intolerance/malabsorption, caused significantly higher expiratory H_2_ values during lactose breath tests. Additionally, these findings may explain why* H. pylori*-infected patients with dyspepsia/unexplained functional, nonspecific, non-allergic GI complaints of the IBS spectrum were considered as a separate group of patients with functional dyspepsia (Schulz and Kupčinskas, 2020[22]).

Stepwise increase of one main symptom of the IBS spectrum, of abdominal pain, was indicated by *H. pylori* patients when this infection is combined with LIT and FM or with LIT, FM and HIT (Figure 2(Fig. 2)). Interestingly, compared to other food intolerance/malabsorption patients, bloating and diarrhea as other main dyspepsia/ functional, nonspecific, non-allergic GI complaints of the IBS spectrum symptoms, were not specified by* H. pylori*-only patients during lactose breath tests. 

In dyspepsia/functional, nonspecific, non-allergic GI complaints patients with food intolerance/malabsorption this suggests mucosal and metabolic differences in *H. pylori* infection caused by varying abilities to digest and/or absorb food components. Moreover, this offers a possibility to explain various phenotypes of LIT. With high end-expiratory H_2_ values during lactose breath tests in *H. pylori *and LIT patients, the probability of additional food intolerance/ malabsorption increases. In *H. pylori* infection and LIT this suggests influence of *H. pylori* and additional food intolerance/malabsorption on digestion.

Acetate, one of the major gut microbial metabolites, increases the production of IgA in the colonic mucosa. It regulates differentially IgA reactivity to commensal bacteria (Takeuchi et al., 2021[25]). However, type, quantity, and composition of food components including dietary carbohydrates and proteins influence the metabolic output of gut microbiota. The presence of lactose in the diet and its influence on the presence of microbiota in the gut is established (Pinto et al., 2021[15]). New findings demonstrate interactions between dietary fructose and host GLUT5 (Glucose-transporter 5) as determinants of the composition of colonic microbiota, and severity of experimental colitis (Basu et al., 2021[1]). We speculate that in *H. pylori* infected patients, with additional FM and LIT, the amount of acetate is influenced. This may explain differences of IgA antibody levels found, as shown in Figure 3(Fig. 3).

In conclusion, we describe, that patients with dyspepsia/unexplained functional, nonspecific, non-allergic GI complaints of the IBS spectrum having LIT and *H. pylori* infection may have additional food intolerance/malabsorption. Moreover, we show that in LIT the presence of *H. pylori* infection causes significantly higher expiratory H_2_ values in lactose tolerance breath tests. Subsequently, besides eradication, a registered and experienced dietician is essential for the development of an individually tailored reduction- or exclusion diet of symptom triggering food components.

## Declaration

### Acknowledgment

We are indebted to Mrs. Katharina Schnedl, Cardiff University, Cardiff, UK, who performed English language corrections.

### Conflict of interest

Wolfgang J. Schnedl received speaking honoraria from Sciotec. The other authors have no conflict of interest. 

## Supplementary Material

Supplementary data

## Figures and Tables

**Table 1:**
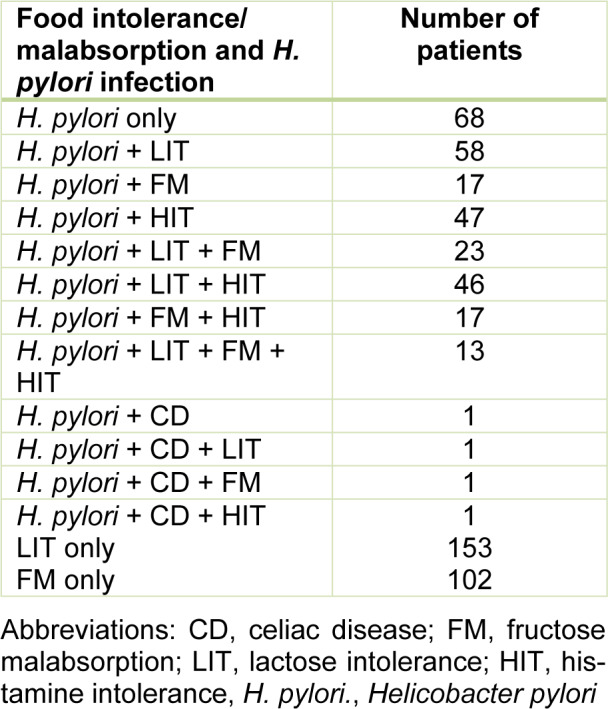
Combinations of *H. pylori* infection and additional food intolerance/malabsorption found in 548 patients with IBS spectrum symptoms, 293 patients had* H. pylori* infection. Separate 153 LIT-only and 102 FM-only patients, without *H. pylori,* infection, were included in evaluations.
