# Supplementary material for: Helicobacter pylori infection and lactose intolerance increase expiratory hydrogen
Source: EXCLI J. 2022 Feb 17;21:426–35. doi: 10.17179/excli2021-4508 (PMC8971348; doi:10.17179/excli2021-4508)
Supplement: Supplementary data [file EXCLI-21-426-s-001.pdf]

EXCLI Journal 2022;21:426-435 – ISSN 1611-2156
Received: November 10, 2021, accepted: February 14, 2022, published: February 17, 2022

Original article:

HELICOBACTER PYLORI INFECTION AND LACTOSE INTOLERANCE INCREASE EXPIRATORY HYDROGEN

Wolfgang J. Schnedl[1], Nathalie Meier-Allard[2], Michael Schenk[3], Sonja Lackner[2], Dietmar Enko[4], Harald Mangge[4], Sandra J. Holasek[2]

[1] Practice for General Internal Medicine, Dr. Theodor Körnerstrasse 19b, A-8600 Bruck/Mur, Austria
[2] Division of Immunology and Pathophysiology, Otto Loewi Research Center, Medical University of Graz, Heinrichstrasse 31a, A-8010 Graz, Austria
[3] Das Kinderwunsch Institut Schenk GmbH, Am Sendergrund 11, A-8143 Dobl, Austria
[4] Clinical Institute of Medical and Chemical Laboratory Diagnosis, Medical University of Graz, Auenbruggerplatz 30, A-8036 Graz, Austria

* **Corresponding author:** Dr. Wolfgang J. Schnedl, Professor of Internal Medicine, Practice for General Internal Medicine, Dr. Theodor Körnerstrasse 19b, A-8600 Bruck/Mur, Austria, Phone: +43-3612-55833, Fax: +43-3612-55833-22, E-mail: w.schnedl@dr-schnedl.at

https://dx.doi.org/10.17179/excli2021-4508

This is an Open Access article distributed under the terms of the Creative Commons Attribution License (http://creativecommons.org/licenses/by/4.0/).

EXCLI Journal 2022;21:426-435 – ISSN 1611-2156
Received: November 10, 2021, accepted: February 14, 2022, published: February 17, 2022

Table 1: Raw data on *H. pylori*-only patients

Year of birth	Gender w (female) m (male)	*H. pylori* (IU/mL)	DAO (IU/mL)	Lactose breath test	Fasting (ppm)	30 Min	60 Min	90 Min	120 Min	Blood glucose fasting	BG 1 (mg%)	BG 2 (mg%)	Symptoms	Fructose breath test	Fasting (ppm)	30 Min	60 Min	90 Min	120 Min	Symptoms	Tissue trans-glutaminase
**1972**	w	pos.	18,4	neg.	2	3	4	9	10	99	131	90	none	neg.	1	3	3	18	11	none	neg.
**1951**	m	pos.	29,7	neg.	1	1	1	1	0	108	132	100	none	neg.	6	4	3	4	3	none	neg.
**1963**	w	pos.	15,8	neg.	1	4	6	4	2	86	124	106	none	neg.	2	1	2	2	2	none	neg
**1967**	w	pos.	16,9	neg.	2	2	1	1	2	109	157	123	abdominal pain	neg.	14	10	9	6	6	none	neg.
**1951**	w	pos.	18,9	neg.	1	2	1	1	1	89	162	121	none	neg.	5	4	2	1	1	none	neg.
**1960**	w	85	27,1	neg.	11	12	9	7	13	84	148	102	none	neg.	7	5	6	4	3	none	neg.
**1949**	m	21	20,2	neg.	1	1	1	1	1	110	169	107	none	neg.	2	9	3	2	5	none	neg.
**1996**	m	28	11,7	neg.	4	4	6	5	7	86	118	81	none	neg.	6	8	4	5	4	none	neg.
**1968**	m	49	42,1	neg.	6	6	5	4	4	123	150	107	rumbling	neg.	2	2	1	1	2	none	neg.
**1940**	w	pos.	37,7	neg.	1	3	1	1	1	92	161	112	none	neg.	1	2	3	12	16	none	neg.
**1953**	w	pos.	28,3	neg.	3	3	3	3	4	114	193	95	none	neg.	1	2	2	2	2	none	neg.
**1968**	m	44	11,8	neg.	4	6	4	3	3	108	160	133	none	neg.	2	3	2	2	2	none	neg.
**1957**	m	42	14,2	neg.	19	20	12	13	15	87	115	95	none	neg.	13	14	11	12	9	none	neg.
**1943**	w	163	24,8	neg.	2	3	2	4	3	108	146	108	none	neg.	3	4	2	3	3	none	neg.
**1955**	w	pos.	22,2	neg.	2	3	1	1	0	66	128	95	none	neg.	2	3	4	4	4	none	neg.
**1955**	w	20	11,4	neg.	0	2	1	0	1	99	187	104	none	neg.	3	3	3	3	2	none	neg.
**1932**	w	pos.	13,5	neg.	14	11	9	8	6	78	128	87	nausea	neg.	4	4	3	5	4	none	neg.
**1964**	m	31	14,9	neg.	2	2	1	1	1	91	118	98	none	neg.	2	3	2	1	1	none	neg.
**1943**	m	22	16,1	neg.	2	2	1	2	2	105	169	118	none	neg.	1	2	8	6	4	none	neg.
**1948**	w	62	15	neg.	2	3	5	6	6	123	145	113	none	neg.	3	3	3	4	5	none	neg.
**1967**	m	pos.	23,8	neg.	1	1	1	3	5	104	115	91	none	neg.	1	3	11	17	16	none	neg.
**1948**	w	pos.	11,5	neg.	8	6	5	6	6	94	124	92	none	neg.	1	1	2	2	2	none	neg.
**1954**	m	pos.	13,4	neg.	0	1	1	1	3	117	154	124	nausea, vertigo	neg.	1	10	19	15	16	none	neg.
**1976**	w	33	17,3	neg.	1	2	1	1	1	107	128	113	none	neg.	1	1	1	1	1	none	neg.

Year of birth	Gender w (female) m (male)	*H. pylori* (IU/mL)	DAO (IU/mL)	Lactose breath test	Fasting (ppm)	30 Min	60 Min	90 Min	120 Min	Blood glucose fasting	BG 1 (mg%)	BG 2 (mg%)	Symptoms	Fructose breath test	Fasting (ppm)	30 Min	60 Min	90 Min	120 Min	Symptoms	Tissue trans-glutaminase
**1957**	w	21	57,8	neg.	4	2	0	0	0	102	126	106	none	neg.	3	2	0	2	2	none	neg.
**1985**	w	26	19,3	neg.	18	19	14	9	9	90	123	89	none	neg.	6	3	2	3	3	abdominal pain, nausea	neg.
**1949**	w	200	10,1	neg.	2	3	2	1	2	98	127	104	none	neg.	2	3	4	6	6	none	neg.
**1956**	w	70	35,8	neg.	2	13	9	5	2	95	128	100	none	neg.	1	1	1	1	1	heartburn	neg.
**1979**	w	200	33,2	neg.	2	2	6	2	2	88	120	101	none	neg.	1	3	10	7	6	rumbling	neg.
**1959**	m	54	20,4	neg.	2	3	3	3	2	85	116	102	none	neg.	4	4	3	4	5	none	neg.
**1944**	m	pos.	36,9	neg.	2	2	1	1	1	103	127	101	none	neg.	3	2	1	1	1	none	neg.
**1970**	m	21	59,7	neg.	2	2	2	2	2	77	105	90	abdominal pain	neg.	5	7	5	7	9	none	neg.
**1977**	m	47	19,3	neg.	8	4	5	4	3	86	119	74	abdominal pain	neg.	3	3	12	7	5	none	neg.
**1990**	m	pos.	15	neg.	1	2	2	2	1	94	119	110	none	neg.	10	9	8	10	14	bloating, nausea	neg.
**1958**	w	153	19	neg.	4	6	8	6	5	117	144	128	none	neg.	1	12	18	8	5	nausea, diarrhea, abdominal pain	neg.
**1947**	w	169	18,8	neg.	1	11	10	13	7	149	208	185	none	neg.	1	7	13	20	14	none	neg.
**1954**	w	30	38	neg.	6	3	5	7	5	188	322	304	none	neg.	2	1	2	4	3	none	neg.
**1955**	w	21	48,1	neg.	1	2	3	2	2	122	186	156	none	neg.	1	7	9	4	6	nausea, diarrhea	neg.
**1986**	w	21	26,8	neg.	9	9	6	5	7	96	137	108	none	neg.	4	5	2	2	2	heartburn	neg.
**1964**	w	69	16,5	neg.	3	4	2	1	1	83	120	85	headache	neg.	2	1	2	3	2	none	neg.
**1958**	m	200	14,4	neg.	2	2	4	4	2	113	143	118	none	neg.	1	3	7	11	12	none	neg.
**1927**	m	43	13,4	neg.	1	3	6	16	11	96	130	94	none	neg.	3	8	14	9	9	none	neg.
**1942**	w	25	38,6	neg.	1	5	2	2	1	110	131	98	stomach pain	neg.	2	1	1	1	1	none	neg.
**1977**	m	31	25,2	neg.	8	8	5	4	4	93	127	114	none	neg.	3	4	3	3	3	none	neg.
**1993**	w	34	14,2	neg.	3	2	2	2	1	93	135	99	none	neg.	15	17	13	15	12	none	neg.
**1947**	w	61	15,9	neg.	3	5	5	7	6	99	148	98	none	neg.	11	9	13	6	5	none	neg.

Year of birth	Gender w (female) m (male)	*H. pylori* (IU/mL)	DAO (IU/mL)	Lactose breath test	Fasting (ppm)	30 Min	60 Min	90 Min	120 Min	Blood glucose fasting	BG 1 (mg%)	BG 2 (mg%)	Symptoms	Fructose breath test	Fasting (ppm)	30 Min	60 Min	90 Min	120 Min	Symptoms	Tissue trans-glutaminase
**1954**	m	44	20,1	neg.	7	3	2	2	2	110	170	134	eczema	neg.	9	12	13	8	6	none	neg.
**1975**	m	157	14,8	neg.	8	4	5	2	3	117	158	101	none	neg.	6	9	5	4	4	none	neg.
**1984**	w	39	14,4	neg.	6	9	7	7	6	93	117	105	headache	neg.	2	3	1	1	1	none	neg.
**1969**	m	22	16,1	neg.	3	6	2	3	2	116	165	113	none	neg.	2	4	3	3	3	none	neg.
**1975**	w	105	59,4	neg.	0	2	2	2	3	107	134	109	none	neg.	1	3	1	1	1	none	neg.
**1964**	w	39	21,5	neg.	2	3	2	3	1	95	162	107	none	neg.	1	5	2	1	1	none	neg.
**1946**	w	36	29,5	neg.	8	6	5	6	4	148	215	224	none	neg.	10	15	14	8	6	none	neg.
**1986**	m	99	21,1	neg.	10	8	3	1	3	135	148	112	abdominal pain	neg.	2	3	4	7	5	none	neg.
**1991**	w	21	23,4	neg.	10	11	13	8	8	94	135	100	none	neg.	2	3	3	2	2	none	neg.
**1979**	m	28	13,4	neg.	8	7	5	3	4	102	165	141	stomach pain	neg.	2	3	7	7	5	none	neg.
**1995**	w	39	16,7	neg.	5	7	7	4	5	111	136	115	nausea, headache	neg.	3	4	6	5	5	none	neg.
**1982**	m	108	17,6	neg.	2	1	1	1	2	127	155	135	none	neg.	11	8	19	15	11	none	neg.
**1984**	w	43	21,9	neg.	6	5	3	3	3	99	150	101	rumbling	neg.	11	16	19	24	30	none	neg.
**1975**	w	132	29,6	neg.	14	13	12	11	18	103	127	87	stomach pain	neg.	16	12	13	14	10	none	neg.
**1960**	w	66	19,7	neg.	4	3	4	3	3	116	173	97	none	neg.	10	9	12	9	8	none	neg.
**1946**	w	94	36,8	neg.	7	12	10	11	9	118	175	124	none	neg.	6	5	5	6	4	none	neg.
**1973**	w	63	57,8	neg.	2	2	2	11	21	94	124	93	none	neg.	3	3	3	5	4	fullness	neg.
**1988**	w	40	24,4	neg.	14	11	9	8	6	95	125	81	none	neg.	2	2	16	11	5	none	neg.
**1970**	w	115	10,4	neg.	2	2	2	2	2	91	142	122	none	neg.	2	2	5	3	3	none	neg.
**1974**	w	200	14,5	neg.	8	9	3	4	3	85	136	99	none	neg.	3	2	2	1	3	none	neg.
**1970**	w	44	29,5	neg.	4	2	3	3	3	87	133	115	nausea	neg.	6	6	4	2	1	none	neg.
**1974**	w	103	14,5	neg.	8	9	3	4	3	85	136	99	none	neg.	3	2	2	1	3	none	neg.
**1967**	w	29	15,1	neg.	0	0	8	4	5	89	114	93	none	neg.	0	0	0	0	0	none	neg.
**1948**	m	142	14,1	neg.	0	0	1	3	0	207	307	315	none	neg.	3	2	0	4	12	none	neg.
**1975**	w	58	14,6	neg.	0	0	0	0	0	91	135	92	none	neg.	0	0	0	0	0	none	neg.

EXCLI Journal 2022;21:426-435 – ISSN 1611-2156
Received: November 10, 2021, accepted: February 14, 2022, published: February 17, 2022

Year of birth	Gender w (female) m (male)	*H. pylori* (IU/mL)	DAO (IU/mL)	Lactose breath test	Fasting (ppm)	30 Min	60 Min	90 Min	120 Min	Blood glucose fasting	BG 1 (mg%)	BG 2 (mg%)	Symptoms	Fructose breath test	Fasting (ppm)	30 Min	60 Min	90 Min	120 Min	Symptoms	Tissue trans-glutaminase
**1940**	m	200	13,5	neg.	0	11	9	3	4	102	133	88	none	neg.	0	8	2	3	3	diarrhea	neg.
**1994**	w	145	10,5	neg.	6	6	2	0	0	75	100	67	none	neg.	8	1	0	0	0	none	neg.
**1968**	m	200	11,7	neg.	4	2	0	0	0	119	171	114	none	neg.	8	5	3	2	1	none	neg.
**1964**	m	26	63,4	neg.	4	9	9	10	6	94	129	94	none	neg.	0	2	5	4	0	none	neg.
**1961**	m	83	13,6	neg.	3	6	5	1	0	112	147	87	rumbling	neg.	4	10	7	0	0	none	neg.
**1944**	w	38	10,9	neg.	0	1	4	0	5	89	137	131	headache	neg.	11	9	24	17	28	none	neg.

Table 2: Raw data on *H. pylori* and lactose intolerance patients

Year of birth	Gender w (female) m (male)	*H. pylori* (IU/mL)	DAO (IU/mL)	Lactose breath test	Fasting (ppm)	30 Min	60 Min	90 Min	120 Min	Blood glucose fasting	BG 1 (mg%)	BG 2 (mg%)	Symptoms	Fructose breath test	Fasting (ppm)	30 Min	60 Min	90 Min	120 Min	Symptoms	Tissue trans-glutaminase
**1941**	m	POS.	17,4	pos.	1	2	12	50	34	75	92	93	none	neg.	2	4	8	10	14	none	neg.
**1975**	w	22	14,8	pos.	1	4	12	49	115	85	98	93	diarrhea, bloating	neg.	4	3	3	2	2	none	neg.
**1965**	w	36	25,3	pos.	3	38	104	167	168	96	115	115	diarrhea, bloating	neg.	5	3	3	2	5	none	neg.
**1998**	w	POS.	19	pos.	17	13	17	33	39	114	102	97	nausea, fatigue	neg.	8	3	4	5	7	none	neg.
**1940**	w	POS.	31	pos.	1	5	21	62	118	97	91	105	none	neg.	1	2	1	1	1	none	neg.
**1979**	m	35	21	pos.	14	20	15	42	80	105	110	106	none	neg.	57	25	18	18	15	none	neg.
**1962**	w	POS.	13,3	pos.	1	5	5	36	110	97	110	103	rumbling, abdominal pain	neg.	9	8	6	7	3	abdominal pain	neg.
**1936**	m	POS.	11,9	pos.	15	34	18	33	46	110	147	116	none	neg.	12	13	18	10	9	none	n.g.
**1961**	m	32	13,1	pos.	6	15	53	100	180	98	127	105	rumbling	neg.	2	10	21	18	11	rumbling	neg.
**1953**	w	64	11,4	pos.	5	20	57	114	200	90	101	105	rumbling	neg.	6	10	20	12	11	rumbling, hick ups	neg.
**1963**	w	26	17,4	pos.	2	3	6	23	30	100	102	100	bloating	neg.	2	3	16	19	5	none	neg.
**1958**	m	29	14	pos.	3	9	56	74	69	101	118	106	nausea	neg.	1	2	2	1	1	none	neg.
**1961**	w	23	18,8	pos.	7	12	10	21	142	130	137	118	none	neg.	5	5	5	3	3	none	neg.
**1973**	w	85	10,5	pos.	1	17	81	161	163	88	111	91	abdominal pain	neg.	2	3	2	2	2	none	neg.
**1979**	m	POS.	15,7	pos.	2	21	62	46	34	110	107	115	rumbling	neg.	9	6	5	4	4	none	neg.
**1977**	w	POS.	20,3	pos.	17	21	20	95	126	95	101	88	none	neg.	7	10	11	7	7	none	neg.
**1970**	m	POS.	16,1	pos.	6	11	38	223	252	104	110	113	none	neg.	3	8	11	12	9	none	neg.
**1940**	w	200	25,4	pos.	1	2	11	24	26	118	147	124	none	neg.	1	3	1	1	1	none	neg.
**1941**	m	200	21,3	pos.	3	8	54	101	107	134	143	125	diarrhea	neg.	14	10	21	19	20	none	neg.
**1962**	m	200	11	pos.	14	10	40	41	150	110	108	100	abdominal pain, diarrhea	neg.	7	7	6	6	3	none	neg.
**1964**	w	67	25,1	pos.	12	11	14	37	106	93	106	82	bloating	neg.	4	8	10	9	6	none	neg.
**1958**	w	20	13,8	pos.	1	35	71	29	24	97	82	95	none	neg.	2	9	16	9	3	none	neg.
**1985**	w	67	21,6	pos.	9	13	9	71	86	77	76	87	none	neg.	6	8	9	7	5	none	neg.
**1984**	m	83	21,8	pos.	0	4	10	43	88	77	102	85	nausea, abdominal pain	neg.	7	5	4	4	3	nausea, vertigo	neg.
**1961**	w	POS.	29,8	pos.	14	20	116	113	228	76	91	77	bloating, rumbling	neg.	5	5	3	6	6	none	neg.
**1966**	m	38	17,4	pos.	4	20	66	65	63	77	89	87	rumbling	neg.	3	2	6	4	2	none	neg.

EXCLI Journal 2022;21:426-435 – ISSN 1611-2156
Received: November 10, 2021, accepted: February 14, 2022, published: February 17, 2022

Year of birth	Gender w (female) m (male)	*H. pylori* (IU/mL)	DAO (IU/mL)	Lactose breath test	Fasting (ppm)	30 Min	60 Min	90 Min	120 Min	Blood glucose fasting	BG 1 (mg%)	BG 2 (mg%)	Symptoms	Fructose breath test	Fasting (ppm)	30 Min	60 Min	90 Min	120 Min	Symptoms	Tissue trans-glutaminase
**1966**	w	POS.	80	pos.	6	7	8	21	64	91	105	97	none	neg.	1	1	1	0	1	diarrhea	neg.
**1995**	w	30	41,4	pos.	1	4	25	42	55	74	98	91	none	neg.	19	11	10	8	7	none	neg.
**1966**	w	POS.	17,2	pos.	13	9	53	77	215	106	104	97	bloating	neg.	3	4	9	8	6	none	neg.
**1979**	w	POS.	13,1	pos.	5	7	44	74	194	86	99	98	adbdominal pain	neg.	10	9	15	17	15	rumbling	neg.
**1965**	w	22	32	pos.	7	7	8	11	64	100	114	110	none	neg.	1	1	1	2	1	itchy skin	neg.
**1979**	w	22	14,5	pos.	3	8	82	95	90	88	117	110	rumbling	neg.	2	8	3	5	6	none	neg.
**1965**	w	POS.	15,2	pos.	3	4	17	17	74	94	93	94	adbdominal pain, diarrhea	neg.	8	12	10	8	2	none	neg.
**1979**	m	55	34,9	pos.	5	3	4	12	59	88	102	93	rumbling	neg.	3	3	5	6	5	none	neg.
**1984**	w	69	12,4	pos.	7	7	13	23	38	97	103	94	bloating	neg.	18	14	16	9	15	none	neg.
**1974**	w	72	16,5	pos.	16	22	14	37	98	91	101	101	rumbling, nausea	neg.	4	5	5	5	3	none	neg.
**1976**	m	42	27,6	pos.	5	5	5	36	52	123	135	116	none	neg.	16	18	12	15	11	none	neg.
**1961**	w	71	13,3	pos.	3	6	28	34	30	94	112	114	none	neg.	1	3	5	2	1	nausea	neg.
**1973**	w	74	22,7	pos.	14	18	29	108	189	100	116	119	none	neg.	16	15	15	13	15	none	neg.
**1979**	m	30	11,1	pos.	13	15	74	83	47	109	123	115	none	neg.	20	22	28	14	16	none	neg.
**1974**	m	200	22,1	pos.	10	22	16	135	116	89	114	92	abdominal pain	neg.	14	17	19	16	7	none	neg.
**1968**	m	70	75,5	pos.	4	14	79	136	127	103	108	91	bloating	neg.	19	19	20	23	15	fullness	neg.
**1971**	w	41	16,8	pos.	4	2	16	23	31	109	139	105	none	neg.	4	1	0	0	0	none	neg.
**1971**	w	140	24	pos.	4	31	93	88	102	156	166	167	heartburn	neg.	5	17	11	8	9	abdominal pain	neg.
**1986**	w	81	17,3	pos.	11	10	34	168	278	104	126	112	abdominal pain, bloating	neg.	6	6	5	20	4	none	neg.
**1935**	m	10	20,2	pos.	0	6	51	117	325	129	128	121	gas, empty stomach	neg.	14	15	0	0	0	none	neg.
**1990**	m	60	23,2	pos.	9	11	10	73	64	114	116	118	abdominal pain, diarrhea	neg.	0	0	0	0	0	vertigo	neg.
**1974**	w	20	11,3	pos.	8	6	5	4	2	95	90	96	none	neg.	13	10	12	7	6	none	neg.
**1957**	w	POS.	26,9	pos.	1	1	0	1	1	116	115	107	abdominal pain	neg.	1	1	1	1	1	abdominal pain	neg.
**1967**	9	POS.	12,2	pos.	3	3	2	2	1	357	312	231	none	neg.	16	19	13	15	11	none	neg.
**1959**	w	POS.	14,2	pos.	2	4	1	1	2	118	102	93	abdominal pain	neg.	4	3	3	4	3	none	neg.
**1982**	m	POS.	13,1	pos.	4	3	2	1	1	94	84	89	abdominal pain	neg.	5	5	4	4	5	none	neg.

EXCLI Journal 2022;21:426-435 – ISSN 1611-2156
Received: November 10, 2021, accepted: February 14, 2022, published: February 17, 2022

Year of birth	Gender w (female) m (male)	*H. pylori* (IU/mL)	DAO (IU/mL)	Lactose breath test	Fasting (ppm)	30 Min	60 Min	90 Min	120 Min	Blood glucose fasting	BG 1 (mg%)	BG 2 (mg%)	Symptoms	Fructose breath test	Fasting (ppm)	30 Min	60 Min	90 Min	120 Min	Symptoms	Tissue trans-glutaminase
**1982**	w	46	11,3	pos.	3	3	3	1	1	112	103	101	none	neg.	2	3	3	5	8	rumbling	neg.
**1965**	w	POS.	11,1	pos.	2	3	10	20	15	93	94	86	none	neg.	4	4	6	9	5	none	neg.
**1972**	w	34	20,7	pos.	8	9	8	8	7	89	81	91	abdominal pain	neg.	1	2	1	1	1	none	neg.
**1970**	w	84	52	pos.	4	4	4	3	4	106	112	109	headache	neg.	2	2	2	1	1	none	neg.
**1977**	m	72	20,6	pos.	20	13	15	7	5	113	123	108	rumbling, diarrhea	neg.	12	7	5	3	2	rumbling	neg.
**1969**	w	126	18,8	pos.	8	8	5	5	4	113	114	110	heartburn	neg.	14	13	12	22	28	abdominal pain, headache	neg.
**1970**	m	POS.	47,7	pos.	11	11	9	9	6	102	106	96	throat mucus	neg.	10	6	7	7	5	none	neg.
**1978**	m	200	37,1	pos.	1	2	1	1	4	110	118	106	abdominal pain	neg.	6	7	4	5	7	abdominal pain	neg.
**1984**	w	25	14,7	pos.	5	8	6	5	3	94	89	86	abdominal pain	neg.	5	4	4	4	1	none	neg.
**1968**	w	POS.	13,6	pos.	0	0	0	0	1	83	80	74	none	neg.	0	0	0	0	0	none	neg.
**1973**	w	36	14,8	pos.	0	0	0	1	1	104	109	87	abdominal fullness	neg.	5	4	2	2	1	none	neg.
**1986**	m	21	13,4	pos.	3	0	7	3	3	99	111	99	none	neg.	8	6	7	5	3	none	neg.
**1967**	w	65	16,1	pos.	3	4	5	5	8	126	121	112	none	neg.	5	7	11	6	1	abdominal pain	neg.
**1970**	m	28	13,5	pos.	20	46	25	44	62	99	108	101	rumbling	neg.	8	14	22	20	8	nausea	neg.

EXCLI Journal 2022;21:426-435 – ISSN 1611-2156
Received: November 10, 2021, accepted: February 14, 2022, published: February 17, 2022

Table 3: Raw data on *H. pylori*-, lactose and histamine intolerant patients

Year of birth	Gender w (female) m (male)	*H. pylori* (IU/mL)	DAO (IU/mL)	Lactose breath test	Fasting (ppm)	30 Min	60 Min	90 Min	120 Min	Blood glucose fasting	BG 1 (mg%)	BG 2 (mg%)	Symptoms	Fructose breath test	Fasting (ppm)	30 Min	60 Min	90 Min	120 Min	Symptoms	Tissue trans-glutaminase
**1984**	w	pos.	9,9	pos.	17	13	11	38	98	91	106	88	none	neg.	4	5	6	7	9	none	neg.
**1993**	w	38	4	pos.	13	9	9	19	30	83	95	80	rumbling	neg.	10	8	9	7	6	none	neg.
**1941**	w	pos.	7,7	pos.	2	12	28	40	122	140	134	142	abdominal pain, bloating	neg.	2	2	2	1	2	none	neg.
**1970**	m	30	8,3	pos.	4	13	30	15	30	101	96	89	diarrhea	neg.	5	23	17	12	11	none	neg.
**1964**	m	200	8,1	pos.	7	7	18	34	30	102	125	134	abdominal pain	neg.	5	13	6	11	9	none	neg.
**1979**	m	pos.	1,5	pos.	0	8	21	57	69	73	93	83	none	neg.	1	2	13	9	9	none	neg.
**1998**	m	23	8	pos.	9	14	5	4	5	110	93	82	none	neg.	15	17	11	11	9	none	neg.
**1985**	w	31	7,7	pos.	17	41	135	45	84	111	107	101	abdominal pain	neg.	11	11	8	10	4	rumbling, abdominal pain	neg.
**1941**	w	22	1,5	pos.	1	1	2	1	1	92	92	105	abdominal pain	neg.	2	10	18	20	15	none	neg.
**1987**	w	20	8,6	pos.	17	22	20	27	46	89	113	80	none	neg.	1	3	13	19	10	stomach pain	neg.
**1981**	w	88	7,2	pos.	1	2	2	5	9	113	101	95	diarrhea	neg.	2	3	3	1	1	none	neg.
**1986**	w	200	6,1	pos.	4	6	6	9	5	104	109	94	none	neg.	2	3	1	7	11	none	neg.
**1969**	w	28	4,7	pos.	4	3	3	3	3	94	104	83	abdominal pain, bloating	neg.	5	4	4	3	2	none	neg.
**1950**	m	42	6,6	pos.	1	8	10	15	23	107	124	77	none	neg.	0	16	18	15	12	none	neg.
**1975**	w	35	6,7	pos.	5	6	19	26	40	84	92	102	none	neg.	11	9	14	10	6	nausea	neg.
**1948**	m	pos.	4,3	pos.	0	1	0	2	4	107	103	112	none	neg.	7	7	15	25	9	none	n.g.
**1981**	m	49	7,1	pos.	8	6	18	21	29	82	102	95	none	neg.	2	5	10	12	10	none	neg.
**1939**	w	pos.	6	pos.	1	2	4	8	15	117	107	102	none	neg.	5	5	9	10	15	none	neg.
**1938**	w	200	8,9	pos.	1	6	20	36	22	93	101	99	none	neg.	3	4	4	7	10	none	neg.
**1986**	w	200	8,7	pos.	17	16	14	11	11	99	105	100	abdominal pain	neg.	9	10	10	9	4	abdominal pain, bloating	neg.

EXCLI Journal 2022;21:426-435 – ISSN 1611-2156
Received: November 10, 2021, accepted: February 14, 2022, published: February 17, 2022

Year of birth	Gender w (female) m (male)	*H. pylori* (IU/mL)	DAO (IU/mL)	Lactose breath test	Fasting (ppm)	30 Min	60 Min	90 Min	120 Min	Blood glucose fasting	BG 1 (mg%)	BG 2 (mg%)	Symptoms	Fructose breath test	Fasting (ppm)	30 Min	60 Min	90 Min	120 Min	Symptoms	Tissue trans-glutaminase
**1987**	w	33	7,3	pos.	15	27	69	162	243	96	109	103	nausea, abdominal pain	neg.	13	17	18	18	10	none	neg.
**1991**	w	28	2	pos.	3	1	2	2	2	101	109	76	stomach pain, bloating	neg.	0	1	1	0	1	none	neg.
**1976**	m	153	6,9	pos.	2	13	80	121	188	93	130	99	abdominal pain, diarrhea	neg.	1	4	5	6	7	none	neg.
**1990**	m	200	5,7	pos.	11	14	72	110	81	103	98	98	rumbling, burping	neg.	9	15	12	14	5	rumbling	neg.
**1960**	w	200	9,9	pos.	3	5	16	12	11	102	99	92	none	neg.	5	7	8	4	3	headache	neg.
**1977**	m	pos.	9,7	pos.	14	10	13	11	5	130	128	92	none	neg.	2	3	3	2	2	none	neg.
**1982**	w	87	7,8	pos.	6	3	2	2	3	115	120	110	fatigue	neg.	4	2	5	4	4	none	neg.
**1990**	w	43	7,5	pos.	7	4	20	70	105	91	109	104	bloating, abdominal pain	neg.	23	13	11	8	7	none	neg.
**1976**	m	110	7,6	pos.	7	11	14	12	33	97	109	103	none	neg.	4	3	3	3	3	none	neg.
**1974**	m	22	8,2	pos.	11	18	10	11	12	109	117	111	none	neg.	4	4	4	4	2	none	neg.
**1981**	m	22	6,4	pos.	4	2	50	77	43	104	111	93	bloating	neg.	3	8	13	15	21	none	neg.
**1976**	m	200	6,6	pos.	18	46	138	157	152	82	152	164	abdominal pain	neg.	16	16	17	13	9	none	neg.
**1958**	m	pos.	1,5	pos.	5	2	15	43	41	126	186	144	none	neg.	10	13	11	6	6	none	neg.
**1938**	w	41	4,2	pos.	2	3	11	30	47	114	118	127	diarrhea	neg.	4	9	11	7	4	none	neg.
**1976**	m	200	6,6	pos.	18	46	138	157	152	82	152	164	abdominal pain	neg.	16	16	17	13	9	none	neg.
**1994**	w	200	3,2	pos.	0	0	25	33	92	87	94	86	abdominal pain	neg.	0	0	0	13	9	none	neg.
**1996**	w	38	5,6	pos.	8	2	33	59	79	89	94	93	diarrhea, abdominal pain	neg.	5	0	0	0	0	none	neg.
**1958**	w	26	6,4	pos.	0	0	0	18	60	88	106	96	none	neg.	9	9	22	24	10	abdominal pain	neg.
**1956**	w	34	5,5	pos.	1	0	0	0	0	116	117	100	none	neg.	0	1	6	8	9	nausea	neg.
**1985**	w	51	2,5	pos.	4	10	5	2	1	103	101	80	none	neg.	2	0	9	1	0	none	neg.

EXCLI Journal 2022;21:426-435 – ISSN 1611-2156
Received: November 10, 2021, accepted: February 14, 2022, published: February 17, 2022

**Year of birth**	**Gender w (female) m (male)**	***H. pylori* (IU/mL)**	**DAO (IU/mL)**	**Lactose breath test**	**Fasting (ppm)**	**30 Min**	**60 Min**	**90 Min**	**120 Min**	**Blood glucose fasting**	**BG 1 (mg%)**	**BG 2 (mg%)**	**Symptoms**	**Fructose breath test**	**Fasting (ppm)**	**30 Min**	**60 Min**	**90 Min**	**120 Min**	**Symptoms**	**Tissue trans-glutaminase**
**1970**	m	41	6,4	pos.	0	14	65	110	138	92	103	71	abdominal pain	neg.	4	4	2	1	0	none	neg.
**1977**	m	37	8,6	pos.	7	7	36	103	235	99	103	93	none	neg.	17	13	9	10	8	none	neg.
**1966**	w	67	9,2	pos.	3	5	203	208	281	92	114	80	bloating, nausea	neg.	8	9	10	24	26	none	neg.
**1977**	w	102	1,6	pos.	3	2	52	59	81	109	98	98	bloating, diarrhea	neg.	12	9	5	7	1	none	neg.
**1983**	m	200	5,4	pos.	8	11	4	13	13	120	111	106	heartburn	neg.	14	0	0	0	0	none	neg.
**1980**	m	79	1,6	pos.	4	18	5	21	39	126	149	142	none	neg.	3	0	0	0	0	none	neg.
**1969**	w	51	7,2	pos.	0	2	4	3	0	99	104	89	headache	neg.	0	0	0	1	4	headache	neg.
**1968**	w	67	6,1	pos.	2	3	3	0	1	77	96	93	abdominal pain	neg.	0	0	0	0	0	none	neg.
**1995**	m	56	5,1	pos.	0	3	1	29	50	118	121	99	abdominal pain, diarrhea	neg.	7	2	0	0	0	none	neg.
**1943**	w	67	9,9	pos.	3	12	10	3	0	109	121	91	none	neg.	10	13	15	8	10	none	neg.
**1985**	w	75	4,7	pos.	11	10	9	10	7	106	101	90	rumbling	neg.	7	6	25	7	1	none	neg.

EXCLI Journal 2022;21:426-435 – ISSN 1611-2156
Received: November 10, 2021, accepted: February 14, 2022, published: February 17, 2022

Table 4: Raw data on *H. pylori*-, lactose-, histamine intolerant patients with fructose malabsorption

Year of birth	Gender w (female) m (male)	*H. pylori* (IU/mL)	DAO (IU/mL)	Lactose breath test	Fasting (ppm)	30 Min	60 Min	90 Min	120 Min	Blood glucose fasting	BG 1 (mg%)	BG 2 (mg%)	Symptoms	Fructose breath test	Fasting (ppm)	30 Min	60 Min	90 Min	120 Min	Symptoms	Tissue trans-glutaminase
**1979**	w	pos.	6,7	pos.	5	42	141	130	139	87	107	122	bloating, abdominal pain	pos.	4	12	43	32	29	rumbling, bloating, belching	neg.
**1962**	w	pos.	5,9	pos.	0	1	1	2	24	101	111	89	stomach pain	pos.	0	4	52	74	52	abdominal pain	neg.
**1960**	w	100	9,9	pos.	2	4	11	14	30	109	137	116	none	pos.	13	20	40	53	49	none	neg.
**1996**	m	101	8,2	pos.	12	27	57	90	71	104	102	104	rumbling, diarrhea	pos.	2	11	48	36	33	none	neg.
**1978**	w	73	9,7	pos.	5	5	5	4	4	102	100	103	none	pos.	4	6	6	39	32	none	neg.
**1983**	w	30	9,2	pos.	10	27	27	41	26	93	98	92	diarrhea, abdominal pain	pos.	5	8	24	27	36	none	neg.
**1997**	w	86	7,8	pos.	16	19	91	109	173	93	102	92	abdominal pain, bloating	pos.	13	36	73	79	61	abdominal pain, bloating	neg.
**1964**	w	194	2,7	pos.	13	36	70	115	110	104	120	109	diarrhea	pos.	8	8	51	13	5	rumbling	neg.
**1963**	w	20	8,3	pos.	1	5	58	209	109	83	118	81	abdominal pain, bloating, headache	pos.	5	21	92	111	22	headache, nausea	neg.
**1980**	w	62	6,9	pos.	0	59	268	271	182	90	96	88	abdominal pain, bloating, diarrhea	pos.	6	15	62	42	17	abdominal pain	neg.
**1937**	w	47	2,1	pos.	0	3	3	28	59	86	164	99	abdominal pain	pos.	0	0	23	68	110	abdominal pain, headache	neg.
**1989**	m	103	9,4	pos.	3	0	1	2	2	112	107	122	vertigo, nausea	pos.	11	5	52	29	53	none	neg.
**1995**	w	200	5,7	pos.	1	0	5	7	26	93	123	93	none	pos.	4	66	113	140	153	abdominal pain	neg.

EXCLI Journal 2022;21:426-435 – ISSN 1611-2156
Received: November 10, 2021, accepted: February 14, 2022, published: February 17, 2022

Table 5: Raw data on *H. pylori* infected patients with fructose malabsorption

Year of birth	Gender w (female) m (male)	*H. pylori* (IU/mL)	DAO (IU/mL)	Lactose breath test	Fasting (ppm)	30 Min	60 Min	90 Min	120 Min	Blood glucose fasting	BG 1 (mg%)	BG 2 (mg%)	Symptoms	Fructose breath test	Fasting (ppm)	30 Min	60 Min	90 Min	120 Min	Symptoms	Tissue trans-glutaminase
**1994**	w	pos.	17,4	neg.	20	16	17	13	9	84	112	71	none	pos.	10	11	46	52	48	none	neg.
**1933**	w	pos.	22,5	neg.	1	9	4	3	6	129	177	135	none	pos.	10	20	26	26	39	abdominal pain, nausea	neg.
**1964**	w	21	10,7	neg.	0	4	3	0	0	88	126	69	none	pos.	0	59	138	46	24	none	neg.
**1946**	w	200	12,9	neg.	2	2	1	3	3	102	148	113	none	pos.	4	45	25	16	19	belching, vertigo	neg.
**1953**	w	40	11,8	neg.	1	2	1	1	2	108	152	105	none	pos.	1	20	29	14	13	nausea, diarrhea, belching	neg.
**1965**	w	46	11,5	neg.	3	2	1	1	1	83	131	99	none	pos.	3	7	37	35	20	bloating	neg.
**1986**	w	93	13	neg.	2	1	3	1	1	99	111	97	none	pos.	1	2	23	18	16	none	neg.
**1961**	w	25	15,9	neg.	10	8	12	9	10	102	166	130	nausea, rumbling	pos.	3	6	39	27	36	rumbling	neg.
**1972**	w	51	17,3	neg.	6	8	11	6	12	134	171	166	none	pos.	8	47	64	69	61	rumbling	neg.
**1957**	w	58	14,6	neg.	2	3	3	7	13	97	149	102	heartburn	pos.	10	23	95	82	105	heartburn, abdominal pain	neg.
**1992**	w	27	27,1	neg.	3	5	17	18	15	132	143	120	none	pos.	5	3	28	15	7	fatigue, diarrhea	neg.
**1975**	w	22	30	neg.	1	3	1	2	2	119	180	125	none	pos.	4	4	32	38	13	headache	neg.
**1942**	w	83	16,2	neg.	1	1	2	1	5	96	149	98	none	pos.	1	9	63	87	83	diarrhea	neg.
**1979**	m	106	59,6	neg.	6	4	3	3	4	104	159	103	none	pos.	14	11	49	41	68	none	neg.
**1998**	w	168	16,9	neg.	10	7	5	4	8	90	145	98	none	pos.	7	22	37	43	43	headache	neg.
**1962**	m	200	30,6	neg.	0	0	0	0	0	102	126	98	none	pos.	4	18	45	26	20	none	neg.
**1965**	w	200	11,7	neg.	11	12	8	6	6	89	117	104	none	pos.	0	45	55	54	68	fatigue, vertigo	neg.
**1968**	m	28	24,8	neg.	2	10	11	9	3	84	123	88	none	pos.	10	3	48	43	18	none	neg.

Year of birth	Gender w (female) m (male)	*H. pylori* (IU/mL)	DAO (IU/mL)	Lactose breath test	Fasting (ppm)	30 Min	60 Min	90 Min	120 Min	Blood glucose fasting	BG 1 (mg%)	BG 2 (mg%)	Symptoms	Fructose breath test	Fasting (ppm)	30 Min	60 Min	90 Min	120 Min	Symptoms	Tissue trans-glutaminase
**1978**	w	34	16,1	neg.	1	3	3	0	0	126	171	109	nausea	pos.	3	10	45	7	13	nausea, diarrhea	neg.
**1964**	m	40	17,4	neg.	5	2	0	2	3	113	135	109	none	pos.	8	12	36	43	67	none	neg.
**1959**	w	38	14,7	neg.	1	2	2	2	1	81	95	104	none	pos.	0	3	28	43	45	headache	neg.
**1957**	w	34	11,7	neg.	5	14	9	4	7	108	161	126	none	pos.	18	14	7	56	74	none	neg.

Table 6: Raw data on *H. pylori* infected patients and histamine intolerance with fructose malabsorption

Year of birth	Gender w (female) m (male)	*H. pylori* (IU/mL)	DAO (IU/mL)	Lactose breath test	Fasting (ppm)	30 Min	60 Min	90 Min	120 Min	Blood glucose fasting	BG 1 (mg%)	BG 2 (mg%)	Symptoms	Fructose breath test	Fasting (ppm)	30 Min	60 Min	90 Min	120 Min	Symptoms	Tissue trans-glutaminase
**1972**	m	pos.	1,6	neg.	3	4	3	2	2	104	155	103	none	pos.	2	7	65	59	12	bloating	neg.
**1971**	w	pos.	1,5	neg.	19	23	20	17	18	77	107	85	none	pos.	17	49	133	94	53	rumbling	neg.
**1968**	m	138	9,2	neg.	4	5	4	3	2	101	146	104	none	pos.	3	4	26	15	6	none	neg.
**1941**	w	pos.	7,3	neg.	7	11	5	5	5	112	178	126	none	pos.	8	15	83	28	10	nausea	neg.
**1966**	m	24	7,1	neg.	2	1	1	1	0	96	164	99	cold fingers	pos.	3	13	27	22	29	bloating, diarrhea	neg.
**1952**	w	196	1,5	neg.	10	17	2	2	0	90	109	83	none	pos.	7	26	78	142	124	rumbling	neg.
**1977**	m	73	2,4	neg.	1	3	2	1	2	93	151	101	none	pos.	6	12	23	33	55	none	neg.
**1992**	w	40	1,5	neg.	7	9	7	6	5	82	97	100	bloating	pos.	22	30	37	46	52	bloating	neg.
**1990**	m	38	10	neg.	7	13	8	8	6	89	111	102	none	pos.	3	10	37	43	15	none	neg.
**1936**	w	40	8,3	neg.	2	7	5	6	13	144	172	159	none	pos.	3	16	19	41	20	belching	neg.
**1958**	w	pos.	4,1	neg.	2	4	3	5	5	93	98	107	none	pos.	3	6	4	21	25	nausea	neg.
**1962**	w	157	3,3	neg.	4	3	4	4	6	106	133	100	none	pos.	10	14	91	90	76	rumbling	neg.
**1942**	m	89	9,8	neg.	0	0	1	3	5	96	190	130	none	pos.	0	16	19	34	28	diarrhea, bloating	neg.
**1976**	w	43	2,8	neg.	1	6	1	1	4	111	124	123	headache	pos.	0	3	42	55	8	itchy skin, headache	neg.
**1949**	w	200	2,3	neg.	4	4	5	5	18	122	140	116	none	pos.	2	16	68	84	58	none	neg.
**1944**	w	53	8,2	neg.	6	12	9	17	9	91	150	119	none	pos.	9	17	44	25	27	vertigo	neg.
**1973**	w	90	4,1	neg.	1	4	1	2	4	86	97	89	headache	pos.	0	6	59	0	0	none	neg.
**1959**	w	pos.	2,6	neg.	5	7	9	2	10	119	149	107	none	pos.	9	3	0	7	73	none	neg.
**1955**	w	49	9,2	neg.	6	10	0	0	3	117	173	127	headache	pos.	0	6	33	21	22	rumbling	neg.
**1969**	w	200	9,7	neg.	7	6	13	4	2	135	161	117	bloating	pos.	13	31	59	57	43	bloating	neg.

EXCLI Journal 2022;21:426-435 – ISSN 1611-2156
Received: November 10, 2021, accepted: February 14, 2022, published: February 17, 2022

Table 7: Raw data on *H. pylori* infected patients with histamine intolerance

Year of birth	Gender w (female) m (male)	*H. pylori* (IU/mL)	DAO (IU/mL)	Lactose breath test	Fasting (ppm)	30 Min	60 Min	90 Min	120 Min	Blood glucose fasting	BG 1 (mg%)	BG 2 (mg%)	Symptoms	Fructose breath test	Fasting (ppm)	30 Min	60 Min	90 Min	120 Min	Symptoms	Tissue trans-glutaminase
**1972**	w	118	3,1	neg.	2	2	2	1	2	80	110	106	none	neg.	2	1	1	1	1	none	neg.
**1966**	m	pos.	9,1	neg.	6	4	3	2	3	130	156	90	none	neg.	3	3	3	3	2	none	neg.
**1950**	m	pos.	2,6	neg.	1	1	1	1	1	142	236	222	none	neg.	3	3	3	4	1	none	neg.
**1940**	w	200	5,3	neg.	1	4	7	6	4	132	205	175	none	neg.	2	4	3	6	5	none	neg.
**1958**	w	pos.	8	neg.	3	3	1	1	1	96	113	92	none	neg.	0	0	1	1	0	none	neg.
**1989**	w	24	9,2	neg.	9	8	5	5	3	96	123	79	none	neg.	17	13	14	24	23	nausea	neg.
**1973**	w	25	2,8	neg.	2	5	6	3	3	83	140	105	none	neg.	0	1	1	0	1	none	neg.
**1929**	w	28	2,5	neg.	1	3	12	15	15	96	108	102	none	neg.	1	2	1	1	1	none	neg.
**1958**	w	85	4,4	neg.	2	1	1	1	1	94	115	110	rumbling	neg.	3	3	2	3	2	belching	neg.
**1973**	m	23	8,8	neg.	0	0	0	0	0	100	120	95	none	neg.	2	1	0	1	1	none	neg.
**1945**	m	102	5,4	neg.	2	8	2	0	12	102	132	116	none	neg.	1	2	3	11	3	none	neg.
**1990**	w	28	2,6	neg.	1	1	2	1	1	80	143	104	none	neg.	1	3	2	2	2	none	neg.
**1952**	w	35	8,1	neg.	3	2	3	3	7	117	159	131	none	neg.	2	4	9	5	2	none	neg.
**1959**	m	pos.	7,8	neg.	1	1	1	3	5	112	166	105	rumbling	neg.	0	5	11	9	6	diarrhea	neg.
**1956**	m	54	1,5	neg.	2	2	1	1	1	96	153	76	none	neg.	8	10	14	17	9	none	neg.
**1969**	w	49	1,5	neg.	4	3	2	2	1	90	132	113	none	neg.	1	1	1	0	1	none	neg.
**1935**	w	34	9,6	neg.	1	1	1	1	1	112	183	156	none	neg.	0	2	1	1	1	none	neg.
**1963**	m	pos.	2	neg.	7	5	9	7	4	89	129	129	none	neg.	12	18	21	18	15	none	neg.
**1967**	w	119	5,8	neg.	1	1	1	1	1	88	144	96	abdominal pain	neg.	1	1	0	1	1	tired	neg.
**1951**	m	36	3,2	neg.	1	5	3	2	2	111	128	83	none	neg.	2	3	5	4	3	none	neg.
**1973**	w	28	7,9	neg.	1	2	2	1	1	98	121	84	none	neg.	5	10	11	11	8	bloating	neg.
**1996**	w	38	3,1	neg.	11	10	4	3	4	82	92	108	none	neg.	3	3	3	3	2	none	neg.
**1979**	w	28	6,5	neg.	2	2	2	2	2	91	134	81	none	neg.	4	3	4	4	3	rumbling	neg.
**1966**	w	72	8,7	neg.	1	2	1	1	1	122	176	160	none	neg.	3	8	19	17	7	none	neg.
**1982**	w	36	9,3	neg.	7	9	5	4	5	84	125	96	abdominal pain, diarrhea	neg.	2	14	12	7	4	none	neg.

Year of birth	Gender w (female) m (male)	*H. pylori* (IU/mL)	DAO (IU/mL)	Lactose breath test	Fasting (ppm)	30 Min	60 Min	90 Min	120 Min	Blood glucose fasting	BG 1 (mg%)	BG 2 (mg%)	Symptoms	Fructose breath test	Fasting (ppm)	30 Min	60 Min	90 Min	120 Min	Symptoms	Tissue trans-glutaminase
**1966**	w	28	1,5	neg.	3	3	2	0	0	110	131	101	none	neg.	7	11	6	2	2	none	neg.
**1976**	m	pos.	8,6	neg.	4	4	2	1	2	91	128	91	none	neg.	1	5	5	4	2	bloating	neg.
**1966**	m	22	1,5	neg.	1	3	3	2	2	83	109	97	none	neg.	5	8	7	3	5	none	neg.
**1966**	w	126	4	neg.	0	1	1	2	1	95	127	104	none	neg.	1	1	1	1	1	nausea	neg.
**1996**	m	25	3,4	neg.	5	3	3	2	2	82	109	51	itchy skin, vertigo	neg.	14	13	11	16	16	itchy skinn	neg.
**1973**	w	28	7,9	neg.	1	2	2	1	1	98	121	84	none	neg.	5	10	11	11	8	none	neg.
**1965**	w	52	8,3	neg.	6	3	3	2	2	96	118	83	none	neg.	7	8	6	10	8	vertigo	neg.
**1972**	w	61	2,2	neg.	2	3	1	1	1	78	147	111	none	neg.	9	16	10	7	4	abdominal pain	neg.
**1973**	w	41	9,7	neg.	8	8	7	7	5	83	131	104	none	neg.	3	3	7	6	5	none	neg.
**1987**	w	26	5,9	neg.	10	12	13	12	8	105	142	131	none	neg.	9	6	5	7	6	none	neg.
**1972**	w	108	6,3	neg.	16	14	11	10	25	127	149	142	none	neg.	6	3	13	7	5	belching, nausea	neg.
**1974**	w	128	4,2	neg.	4	2	7	2	1	89	161	124	none	neg.	5	3	2	3	2	none	neg.
**1965**	w	pos.	2,8	neg.	4	3	5	7	6	107	161	119	nausea, abdominal misfeeling	neg.	7	8	10	11	11	rumbling	neg.
**1996**	w	32	5	neg.	17	18	26	25	27	98	112	105	nausea	neg.	15	20	14	21	14	stomach misfeeling	neg.
**1939**	w	25	9,2	neg.	6	5	3	2	2	103	182	122	none	neg.	8	12	28	11	5	none	neg.
**1984**	m	23	6,8	neg.	3	2	1	1	2	106	144	117	none	neg.	10	8	7	7	8	none	neg.
**1997**	w	200	5,1	neg.	12	12	11	9	10	98	125	121	none	neg.	2	3	2	4	8	none	neg.
**1981**	w	56	5,9	neg.	2	3	2	1	2	98	121	110	fatigue	neg.	2	2	2	3	3	none	neg.
**1974**	w	30	2	neg.	12	11	8	4	5	80	113	91	none	neg.	4	2	3	2	3	nausea, headache	neg.
**1937**	w	200	4,5	neg.	0	0	0	0	0	105	151	106	none	neg.	0	0	9	9	0	none	neg.
**1982**	m	80	9	neg.	3	8	6	6	7	86	110	102	none	neg.	17	22	23	27	21	none	neg.
**1954**	w	66	4,5	neg.	4	5	7	5	4	92	132	101	none	neg.	2	0	0	4	13	none	neg.
**1948**	w	200	4,2	neg.	0	0	1	1	5	238	315	280	bloating, belching	neg.	1	3	4	2	0	none	neg.
**1964**	m	40	2,7	neg.	0	0	0	0	0	94	151	81	belching	neg.	1	0	0	0	0	belching	neg.

Year of birth	Gender w (female) m (male)	*H. pylori* (IU/mL)	DAO (IU/mL)	Lactose breath test	Fasting (ppm)	30 Min	60 Min	90 Min	120 Min	Blood glucose fasting	BG 1 (mg%)	BG 2 (mg%)	Symptoms	Fructose breath test	Fasting (ppm)	30 Min	60 Min	90 Min	120 Min	Symptoms	Tissue trans-glutaminase
**1970**	w	132	9,1	neg.	0	0	0	0	0	97	138	88	bloating	neg.	0	0	0	0	0	none	neg.
**1972**	w	42	3,4	neg.	0	0	0	1	1	93	140	88	none	neg.	0	0	0	0	0	none	neg.
**1968**	w	28	7	neg.	10	10	10	6	2	81	101	74	none	neg.	8	3	2	0	0	none	neg.
**1980**	w	31	6,9	neg.	1	3	18	9	9	93	123	87	none	neg.	8	8	4	4	3	none	neg.
**1955**	w	42	2,1	neg.	9	8	8	6	7	104	147	97	none	neg.	10	3	3	3	19	none	neg.

EXCLI Journal 2022;21:426-435 – ISSN 1611-2156
Received: November 10, 2021, accepted: February 14, 2022, published: February 17, 2022

Table 8: Raw data on *H. pylori*-, lactose intolerant patients with fructose malabsorption

Year of birth	Gender w (female) m (male)	*H. pylori* (IU/mL)	DAO (IU/mL)	Lactose breath test	Fasting (ppm)	30 Min	60 Min	90 Min	120 Min	Blood glucose fasting	BG 1 (mg%)	BG 2 (mg%)	Symptoms	Fructose breath test	Fasting (ppm)	30 Min	60 Min	90 Min	120 Min	Symptoms	Tissue trans-glutaminase
**1975**	w	200	71,00	pos.	0	174	97	156	270	84	98	80	bloating, abdominal pain	pos.	0	12	38	15	17	bloating, abdominal pain	neg.
**1939**	w	pos.	15,50	pos.	10	9	7	7	8	104	98	67	none	pos.	2	1	7	29	10	diarrhea	neg.
**1986**	w	pos.	16,60	pos.	2	1	10	90	51	95	87	79	none	pos.	5	5	12	27	25	bloating	neg.
**1959**	w	200	18,00	pos.	2	3	2	28	114	74	95	97	bloating, nausea	pos.	2	11	29	41	60	nausea, diarrhea, bloating	neg.
**1984**	m	pos.	16,40	pos.	6	6	37	108	83	88	105	103	rumbling	pos.	7	10	23	32	15	vertigo	neg.
**1970**	m	84	12,70	pos.	13	9	7	7	9	112	93	79	heartburn	pos.	8	14	30	26	20	none	neg.
**1950**	m	34	19,90	pos.	3	30	118	133	74	149	201	200	diarrhea	pos.	2	12	74	109	78	diarrhea	neg.
**1958**	w	65	15,70	pos.	7	9	34	77	59	98	106	95	bloating, rumbling	pos.	9	19	42	48	34	none	neg.
**1941**	m	52	17,50	pos.	3	5	16	8	15	108	114	93	none	pos.	2	14	14	46	49	none	neg.
**1976**	w	34	12,60	pos.	4	4	4	3	2	93	86	88	none	pos.	2	9	31	21	18	none	neg.
**1962**	w	48	21,00	pos.	6	9	8	14	19	102	111	110	none	pos.	4	24	36	25	20	none	neg.
**1961**	w	98	20,60	pos.	12	92	144	177	126	100	110	97	abdominal pain	pos.	7	32	40	20	12	none	neg.
**1950**	m	124	19,90	pos.	3	30	118	133	74	149	201	200	diarrhea	pos.	2	12	74	109	78	diarrhea	neg.
**2002**	w	200	12,40	pos.	8	37	75	70	106	90	106	91	none	pos.	10	8	33	44	23	rumbling	neg.
**1983**	m	164	17,00	pos.	31	28	50	72	133	102	101	107	none	pos.	33	71	45	119	101	rumbling	neg.
**1965**	w	36	13,10	pos.	6	5	6	29	46	85	111	117	bloating	pos.	5	13	55	27	23	none	neg.
**1967**	w	40	30,90	pos.	4	7	25	29	18	89	166	155	none	pos.	6	25	68	101	30	none	neg.
**1995**	m	200	13,20	pos.	41	141	215	128	125	98	101	101	diarrhea	pos.	11	23	30	26	23	none	neg.

EXCLI Journal 2022;21:426-435 – ISSN 1611-2156
Received: November 10, 2021, accepted: February 14, 2022, published: February 17, 2022

Year of birth	Gender w (female) m (male)	*H. pylori* (IU/mL)	DAO (IU/mL)	Lactose breath test	Fasting (ppm)	30 Min	60 Min	90 Min	120 Min	Blood glucose fasting	BG 1 (mg%)	BG 2 (mg%)	Symptoms	Fructose breath test	Fasting (ppm)	30 Min	60 Min	90 Min	120 Min	Symptoms	Tissue trans-glutaminase
**1964**	w	200	28,80	pos.	1	3	0	1	0	98	107	91	none	pos.	3	4	10	24	28	headache	neg.
**1981**	w	pos.	22,70	pos.	7	107	239	288	358	98	100	90	headache	pos.	0	14	23	11	9	none	neg.
**1970**	w	pos.	14,7	pos.	10	16	13	13	14	85	101	91	none	pos.	13	17	14	16	46	bloating	n.g.
**1975**	w	200	13,2	pos.	1	3	3	2	1	97	112	109	none	pos.	7	1	6	17	27	abdominal pain	n.g
**2003**	w	40	28,20	pos.	12	4	0	5	0	109	120	115	none	pos.	37	17	32	33	60	none	neg.
**1979**	w	90	12,80	pos.	4	3	37	23	45	113	161	150	abdominal pain, bloating, headache	pos.	8	51	88	77	71	headache, nausea, belching	neg.
**1981**	m	pos.	35,10	pos.	10	16	41	45	37	113	125	89	eczema	pos.	3	3	43	22	23	none	neg.

EXCLI Journal 2022;21:426-435 – ISSN 1611-2156
Received: November 10, 2021, accepted: February 14, 2022, published: February 17, 2022

Table 9: Raw data on lactose intolerant patients

Year of birth	Gender w (female) m (male)	*H. pylori* (IU/mL)	DAO (IU/mL)	Lactose breath test	Fasting (ppm)	30 Min	60 Min	90 Min	120 Min	Blood glucose fasting	BG 1 (mg%)	BG 2 (mg%)	Symptoms	Fructose breath test	Fasting (ppm)	30 Min	60 Min	90 Min	120 Min	Symptoms	Tissue trans-glutaminase
**1983**	m	neg.	34,1	pos.	9	9	7	7	6	117	120	105	none	neg.	6	5	4	4	2	none	neg.
**1953**	w	neg.	18,9	pos.	5	10	20	44	65	131	148	130	bloating	neg.	4	7	5	3	2	none	neg
**1956**	m	neg.	18,4	pos.	9	10	56	115	90	124	132	137	none	neg.	3	3	2	2	1	none	neg.
**1990**	m	neg.	15.1	pos.	8	3	2	1	1	116	117	101	none	neg.	5	3	8	7	3	none	neg.
**1993**	m	neg.	10,2	pos.	5	2	2	3	3	115	94	94	fullness	neg.	1	1	1	1	1	none	neg.
**1970**	w	neg.	38,1	pos.	3	40	83	71	71	108	99	123	stomach pain, bloating	neg.	10	23	17	29	12	bloating, abdominal pain	neg.
**1969**	w	neg.	22,1	pos.	0	1	2	21	22	123	140	138	rumbling	neg.	7	7	9	8	8	none	neg.
**1951**	m	neg.	21,2	pos.	6	5	17	24	31	101	126	78	none	neg.	2	4	15	7	7	none	neg.
**1964**	m	neg.	21,9	pos.	7	4	7	3	46	103	98	96	none	neg.	7	8	3	2	3	none	neg.
**1968**	m	neg.	20,9	pos.	1	17	36	42	55	92	107	106	none	neg.	2	2	3	1	2	none	neg.
**1969**	w	neg.	20,3	pos.	3	3	10	50	76	140	126	121	bloating	neg.	11	10	13	9	11	none	neg.
**1955**	m	neg.	10,5	pos.	5	9	17	53	66	99	108	97	none	neg.	2	7	7	5	2	none	neg.
**1985**	w	neg.	63,7	pos.	4	10	5	8	26	83	104	74	none	neg.	4	4	2	2	2	none	neg
**1972**	w	neg.	12	pos.	7	6	22	50	67	80	102	95	diarrhea, bloating	neg.	9	6	5	4	5	none	neg.
**1985**	m	neg.	17,8	pos.	3	100	66	89	106	101	115	96	bloating	neg.	6	13	22	12	4	none	neg.
**1959**	m	neg.	21,6	pos.	2	23	43	42	67	125	130	130	rumbling	neg.	5	8	11	17	17	none	neg.
**1956**	w	neg.	18	pos.	3	4	4	9	39	123	126	109	abdominal pain	neg.	3	4	3	2	2	bloating	neg.
**1967**	m	neg.	12,1	pos.	5	8	48	66	104	97	104	111	bloating	neg.	11	9	9	8	10	none	neg.
**1942**	m	neg.	34,7	pos.	6	8	8	38	60	111	115	108	bloating	neg.	2	3	2	2	3	none	neg.
**1997**	m	neg.	18,6	pos.	10	12	57	42	67	84	100	96	none	neg.	7	6	7	10	17	none	neg.
**1969**	w	neg.	15,6	pos.	3	6	6	12	26	87	101	80	none	neg.	6	7	4	10	8	none	neg.
**1971**	w	neg.	26,9	pos.	1	1	1	18	46	97	104	97	none	neg.	1	2	1	1	1	nausea	neg.

Year of birth	Gender w (female) m (male)	*H. pylori* (IU/mL)	DAO (IU/mL)	Lactose breath test	Fasting (ppm)	30 Min	60 Min	90 Min	120 Min	Blood glucose fasting	BG 1 (mg%)	BG 2 (mg%)	Symptoms	Fructose breath test	Fasting (ppm)	30 Min	60 Min	90 Min	120 Min	Symptoms	Tissue trans-glutaminase
**1991**	w	neg.	21,2	pos.	9	20	87	107	62	85	92	87	abdominal pain, bloating	neg.	2	2	2	1	2	none	neg.
**1973**	w	neg.	18,4	pos.	1	9	26	55	160	103	90	109	fullness, heartburn, bloating	neg.	11	10	9	6	5	none	neg.
**1959**	w	neg.	13,1	pos.	2	1	11	66	80	94	102	107	none	neg.	1	2	1	1	1	none	neg.
**1996**	w	neg.	31,8	pos.	6	3	67	84	140	89	109	98	rumbling	neg.	2	4	11	7	5	none	neg.
**1991**	w	neg.	11,4	pos.	9	11	20	61	152	101	99	100	rumbling, belching, bloating	neg.	10	11	10	7	7	none	neg.
**1971**	w	neg.	30,5	pos.	2	0	16	13	30	79	98	88	headache	neg.	2	1	5	7	4	none	neg.
**1958**	w	neg.	13,8	pos.	1	35	71	29	24	97	82	95	none	neg.	2	9	16	9	3	diarrhea	neg.
**1952**	w	neg.	22,7	pos.	9	10	8	140	116	101	93	96	none	neg.	2	3	2	1	1	none	neg.
**1954**	w	neg.	15,5	pos.	2	3	9	23	50	88	114	126	none	neg.	1	1	1	1	1	none	neg.
**1936**	w	neg.	44,2	pos.	1	2	19	30	24	87	106	99	diarrhea, stomach pain	neg.	1	6	8	6	4	none	neg.
**1951**	m	neg.	62,5	pos.	3	5	21	168	200	102	109	91	bloating	neg.	4	5	5	4	17	none	n.g.
**1968**	m	neg.	26,6	pos.	22	18	41	54	72	149	158	152	strange stomach feeling	neg.	13	17	9	10	9	none	neg.
**1940**	w	neg.	25,4	pos.	1	2	11	24	26	118	147	124	none	neg.	1	3	1	1	1	none	neg.
**1974**	w	neg.	12	pos.	2	3	10	34	45	99	86	85	abdominal pain	neg.	2	2	3	7	5	none	neg.
**1954**	w	neg.	16,7	pos.	7	93	116	118	219	85	87	101	abdominal pain	neg.	1	10	21	18	16	none	neg.
**1986**	w	neg.	16,2	pos.	1	6	15	35	39	78	99	99	belching	neg.	2	4	2	1	1	none	neg.
**1959**	w	neg.	15,4	pos.	1	16	82	86	59	98	107	106	none	neg.	1	10	13	15	7	none	neg.
**1963**	w	neg.	20,7	pos.	10	17	53	65	76	95	114	102	diarrhea, fullness	neg.	7	6	18	24	18	none	neg.
**1967**	w	neg.	11,2	pos.	4	3	2	7	39	88	103	103	none	neg.	2	3	2	1	1	none	neg.
**1976**	m	neg.	15	pos.	1	2	35	40	38	86	107	118	rumbling	neg.	10	8	5	4	3	none	neg.
**1987**	w	neg.	16,9	pos.	7	9	28	23	29	89	112	95	rumbling	neg.	2	2	3	2	2	none	neg.

EXCLI Journal 2022;21:426-435 – ISSN 1611-2156
Received: November 10, 2021, accepted: February 14, 2022, published: February 17, 2022

Year of birth	Gender w (female) m (male)	*H. pylori* (IU/mL)	DAO (IU/mL)	Lactose breath test	Fasting (ppm)	30 Min	60 Min	90 Min	120 Min	Blood glucose fasting	BG 1 (mg%)	BG 2 (mg%)	Symptoms	Fructose breath test	Fasting (ppm)	30 Min	60 Min	90 Min	120 Min	Symptoms	Tissue trans-glutaminase
**1963**	m	neg.	10,4	pos.	1	5	14	21	45	107	137	99	none	neg.	10	11	11	7	3	none	neg.
**1991**	w	neg.	16,1	pos.	9	14	31	61	61	85	111	97	rumbling	neg.	7	7	10	6	3	none	neg.
**1984**	w	neg.	20,3	pos.	6	5	7	8	62	102	117	102	stomach pain	neg.	4	3	6	9	6	none	neg.
**1990**	m	neg.	13	pos.	5	8	7	6	28	96	114	109	none	neg.	16	13	20	24	13	none	neg.
**1998**	m	neg.	13,8	pos.	12	20	50	85	59	93	104	95	bloating, abdominal pain	neg.	4	11	15	11	8	none	neg.
**1945**	w	neg.	16,1	pos.	1	2	3	42	61	106	117	107	rumbling, bloating	neg.	4	3	3	3	3	none	neg.
**1986**	w	neg.	11,2	pos.	5	36	127	58	58	89	98	100	rumbling, bloating	neg.	5	5	5	4	3	none	neg.
**1988**	w	neg.	65,7	pos.	14	16	15	20	48	82	93	83	headache, abdominal pain	neg.	7	6	3	3	3	none	neg.
**1936**	w	neg.	22,4	pos.	2	5	27	56	123	130	164	176	nausea	neg.	1	3	6	4	2	none	neg.
**1996**	m	neg.	20,6	pos.	0	5	31	34	65	87	85	71	stomach pain	neg.	7	10	11	9	12	none	neg.
**1964**	m	neg.	16,3	pos.	7	7	9	6	40	100	129	107	none	neg.	7	6	4	5	5	none	neg.
**1975**	w	neg.	24,8	pos.	3	163	88	179	188	98	105	117	rumbling, diarrhea	neg.	13	14	24	16	9	none	neg.
**1976**	m	neg.	12,2	pos.	1	1	5	11	30	124	133	127	rumbling	neg.	2	2	2	2	2	none	neg.
**1973**	w	neg.	18,8	pos.	6	9	97	108	243	108	105	104	rumbling, abdominal pain	neg.	4	7	8	5	4	none	neg.
**1967**	m	neg.	10,7	pos.	5	6	15	16	45	95	129	101	stomach pain	neg.	4	4	4	3	2	none	neg.
**1983**	m	neg.	13,5	pos.	2	8	46	60	97	99	119	111	abdominal pain	neg.	2	3	7	7	10	none	neg.
**1940**	m	neg.	37,3	pos.	3	5	33	30	28	83	135	115	diarrhea	neg.	5	5	9	4	2	none	neg.
**1939**	w	neg.	13,8	pos.	11	6	9	29	35	105	115	100	rumbling, diarrhea	neg.	3	7	10	8	6	diarrhea	neg.
**1980**	w	neg.	13,7	pos.	12	8	9	31	36	95	88	95	rumbling, diarrhea, headache	neg.	10	10	11	12	9	headache	neg.

EXCLI Journal 2022;21:426-435 – ISSN 1611-2156
Received: November 10, 2021, accepted: February 14, 2022, published: February 17, 2022

Year of birth	Gender w (female) m (male)	*H. pylori* (IU/mL)	DAO (IU/mL)	Lactose breath test	Fasting (ppm)	30 Min	60 Min	90 Min	120 Min	Blood glucose fasting	BG 1 (mg%)	BG 2 (mg%)	Symptoms	Fructose breath test	Fasting (ppm)	30 Min	60 Min	90 Min	120 Min	Symptoms	Tissue trans-glutaminase
**1996**	w	neg.	21,4	pos.	3	30	156	131	159	92	105	95	bloating, nausea	neg.	14	11	11	21	28	nausea, abdominal pain	neg.
**1996**	w	neg.	10,8	pos.	4	4	3	10	60	124	150	98	abdominal pain	neg.	2	3	2	2	4	none	neg.
**1962**	w	neg.	21,5	pos.	3	24	110	112	102	129	146	122	bloating, rumbling	neg.	4	3	4	5	3	none	neg.
**1943**	w	neg.	17	pos.	2	4	6	24	110	121	133	121	stomach pain, rumbling	neg.	2	4	18	15	9	none	neg.
**1979**	w	neg.	11,4	pos.	5	5	27	73	74	104	114	120	abdominal pain	neg.	14	16	20	18	16	stomach pain	neg.
**1979**	m	neg.	19,2	pos.	8	8	7	8	35	93	96	90	rumbling	neg.	15	10	8	8	6	belching	neg.
**1977**	m	neg.	32,7	pos.	23	32	33	66	60	116	128	111	abdominal pain, diarrhea	neg.	15	10	11	11	10	none	neg.
**1967**	w	neg.	10,9	pos.	17	16	40	85	101	103	112	106	stomach pain	neg.	5	6	7	4	5	none	neg.
**1991**	m	neg.	23,3	pos.	5	8	15	60	112	100	127	106	bloating, rumbling, diarrhea	neg.	10	9	12	10	11	none	neg.
**1990**	m	neg.	17,3	pos.	12	67	77	103	91	104	116	107	rumbling, bloating	neg.	1	1	1	1	1	none	neg.
**1963**	m	neg.	61,7	pos.	1	2	33	59	47	86	100	107	stomach pain	neg.	2	10	11	7	4	none	neg.
**1964**	w	neg.	14,1	pos.	15	11	7	20	41	123	137	118	diarrhea, bloating, throat mucus	neg.	13	9	11	12	11	none	neg.
**1941**	w	neg.	13,7	pos.	1	10	14	13	50	142	155	156	headache	neg.	5	7	7	3	2	headache	neg.
**1961**	w	neg.	28,6	pos.	1	1	22	41	43	86	110	112	bloating	neg.	11	7	9	7	6	none	neg.
**1999**	m	neg.	13,6	pos.	11	10	9	34	98	93	119	114	none	neg.	5	8	8	6	9	none	neg.
**1994**	m	neg.	16,9	pos.	5	5	9	29	103	85	94	101	bloating	neg.	2	3	3	3	3	none	neg.
**1986**	w	neg.	11,3	pos.	13	25	73	90	62	79	105	89	stomach pain, bloating, rumbling	neg.	4	3	3	3	2	nausea	neg.
**1985**	w	neg.	23,6	pos.	10	9	7	12	64	94	110	106	none	neg.	11	15	12	10	11	none	neg.

EXCLI Journal 2022;21:426-435 – ISSN 1611-2156
Received: November 10, 2021, accepted: February 14, 2022, published: February 17, 2022

Year of birth	Gender w (female) m (male)	*H. pylori* (IU/mL)	DAO (IU/mL)	Lactose breath test	Fasting (ppm)	30 Min	60 Min	90 Min	120 Min	Blood glucose fasting	BG 1 (mg%)	BG 2 (mg%)	Symptoms	Fructose breath test	Fasting (ppm)	30 Min	60 Min	90 Min	120 Min	Symptoms	Tissue trans-glutaminase
**1964**	w	neg.	16,2	pos.	10	7	20	52	64	94	104	96	abdominal pain	neg.	10	9	9	6	6	none	neg.
**1962**	w	neg.	24,8	pos.	3	4	44	88	143	106	114	111	diarrhea	neg.	8	10	17	10	8	none	neg.
**1989**	w	neg.	20,8	pos.	0	0	41	112	124	84	97	87	rumbling	neg.	4	2	0	0	0	none	neg.
**1981**	w	neg.	27,8	pos.	11	6	7	22	32	105	182	109	none	neg.	20	10	14	4	1	headache	neg.
**1951**	m	neg.	23,1	pos.	11	59	23	7	0	108	116	97	hunger	neg.	3	4	5	0	0	rumbling	neg.
**1971**	m	neg.	15,6	pos.	0	3	11	25	31	99	107	112	bloating, rumbling	neg.	2	2	1	2	0	none	neg.
**2005**	w	neg.	10,7	pos.	2	3	12	66	57	101	86	90	abdominal pain	neg.	2	8	15	10	1	none	neg.
**1966**	w	neg.	13,2	pos.	0	1	19	65	59	105	105	95	bloating	neg.	0	0	0	0	0	none	neg.
**1960**	m	neg.	20,2	pos.	0	2	2	15	37	107	133	105	none	neg.	4	1	4	11	7	none	neg.
**1997**	w	neg.	17,8	pos.	6	8	13	77	70	95	84	102	headache, abdominal pain, rumbling	neg.	10	9	7	7	4	none	neg.
**1980**	w	neg.	12,8	pos.	0	3	8	65	159	76	102	91	bloating	neg.	5	17	12	7	12	none	neg.
**1991**	m	neg.	16,6	pos.	10	13	11	72	123	95	85	75	bloating	neg.	15	9	5	3	3	none	neg.
**1992**	m	neg.	10,6	pos.	0	12	55	69	60	96	98	89	abdominal pain	neg.	2	3	4	2	4	none	neg.
**1965**	w	neg.	15,6	pos.	13	12	29	52	46	96	135	88	bloating	neg.	13	8	16	6	22	none	neg.
**1977**	m	neg.	20,1	pos.	13	15	30	79	72	108	111	112	none	neg.	5	22	8	4	3	abdominal pain	neg.
**1988**	w	neg.	11,9	pos.	9	0	3	32	75	99	97	98	abdominal pain, vertigo	neg.	2	4	4	4	4	vertigo	neg.
**1999**	m	neg.	24,2	pos.	3	0	73	315	129	90	122	101	vertigo, tired	neg.	8	0	0	0	0	none	neg.
**1972**	m	neg.	24	pos.	1	1	3	0	0	99	105	110	none	neg.	2	2	2	1	1	none	neg.
**1988**	w	neg.	20,6	pos.	7	4	3	5	4	86	83	75	none	neg.	6	8	6	4	4	none	neg
**1979**	w	neg.	10,9	pos.	2	2	3	2	5	103	104	90	bloating	neg.	5	4	2	5	1	bloating, tired	neg.
**1979**	w	neg.	21,4	pos.	3	3	2	2	2	82	92	92	none	neg.	3	4	11	16	12	none	neg.
**1969**	m	neg.	22,5	pos.	3	3	1	1	1	110	107	106	none	neg.	5	4	6	4	5	none	neg.

EXCLI Journal 2022;21:426-435 – ISSN 1611-2156
Received: November 10, 2021, accepted: February 14, 2022, published: February 17, 2022

Year of birth	Gender w (female) m (male)	*H. pylori* (IU/mL)	DAO (IU/mL)	Lactose breath test	Fasting (ppm)	30 Min	60 Min	90 Min	120 Min	Blood glucose fasting	BG 1 (mg%)	BG 2 (mg%)	Symptoms	Fructose breath test	Fasting (ppm)	30 Min	60 Min	90 Min	120 Min	Symptoms	Tissue trans-glutaminase
**1968**	w	neg.	18,6	pos.	6	4	3	2	4	89	106	104	none	neg.	2	1	1	1	1	none	neg.
**1990**	w	neg.	19,2	pos.	3	3	2	2	5	82	92	98	none	neg.	11	18	7	5	3	belching, bloating	neg.
**1990**	w	neg.	21,2	pos.	10	6	6	6	4	93	95	90	none	neg.	7	9	8	9	13	stomach pain	neg.
**1987**	w	neg.	10,2	pos.	5	4	7	5	6	95	96	82	nausea	neg.	4	6	3	2	1	nausea	neg.
**1965**	w	neg.	14	pos.	4	5	2	3	2	93	99	98	none	neg.	3	3	7	4	1	none	neg.
**1964**	w	neg.	15,4	pos.	0	1	2	1	1	105	115	92	abdominal pain, headache	neg.	4	3	3	2	2	none	neg.
**1988**	w	neg.	13,5	pos.	3	4	3	3	2	99	103	85	diarrhea, bloating	neg.	15	19	14	10	6	none	n.g.
**1957**	m	neg.	17,3	pos.	2	7	11	6	13	118	127	124	none	neg.	4	7	4	2	2	diarrhea	neg.
**1985**	w	neg.	12,7	pos.	4	4	3	2	4	77	86	84	none	neg.	4	4	4	4	3	none	neg.
**1988**	w	neg.	14,2	pos.	3	3	7	21	23	103	117	107	none	neg.	3	4	3	4	3	none	neg.
**1987**	m	neg.	18,7	pos.	4	3	2	3	2	105	108	95	none	neg.	13	16	14	12	7	none	neg.
**1975**	m	neg.	12,4	pos.	5	5	3	2	2	96	105	100	none	neg.	1	1	1	1	1	none	neg.
**1990**	w	neg.	12,8	pos.	2	2	1	2	1	83	89	93	none	neg.	5	5	5	4	3	none	neg.
**1948**	m	neg.	10,6	pos.	2	3	2	3	3	105	112	102	none	neg.	6	10	7	6	4	none	neg.
**1995**	w	neg.	24,1	pos.	2	2	13	21	20	89	89	90	belching	neg.	4	3	3	10	13	none	neg.
**1971**	m	neg.	16,2	pos.	5	3	3	12	15	96	101	105	abdominal pain	neg.	2	2	2	1	1	none	neg.
**1982**	m	neg.	11	pos.	3	5	5	3	2	113	115	95	none	neg.	3	2	3	3	2	none	neg.
**1991**	w	neg.	38,3	pos.	11	12	9	14	12	87	87	97	tired, abdominal pain	neg.	15	13	13	15	13	tired, nausea	neg.
**1985**	w	neg.	24,2	pos.	3	3	2	2	2	92	95	97	abdominal pain	neg.	9	12	9	11	7	none	neg.
**1958**	w	neg.	14,3	pos.	3	2	2	2	1	96	106	102	bloating, diarrhea	neg.	3	2	3	4	4	none	neg.
**1997**	w	neg.	20	pos.	8	4	5	4	5	106	107	98	nausea	neg.	10	15	20	14	18	none	neg.
**1985**	m	neg.	15,8	pos.	1	2	1	2	2	105	97	113	none	neg.	6	10	7	6	6	none	neg.
**1964**	m	neg.	24,9	pos.	3	2	2	4	21	113	117	101	none	neg.	1	2	1	1	1	none	neg.

EXCLI Journal 2022;21:426-435 – ISSN 1611-2156
Received: November 10, 2021, accepted: February 14, 2022, published: February 17, 2022

**Year of birth**	**Gender w (female) m (male)**	***H. pylori* (IU/mL)**	**DAO (IU/mL)**	**Lactose breath test**	**Fasting (ppm)**	**30 Min**	**60 Min**	**90 Min**	**120 Min**	**Blood glucose fasting**	**BG 1 (mg%)**	**BG 2 (mg%)**	**Symptoms**	**Fructose breath test**	**Fasting (ppm)**	**30 Min**	**60 Min**	**90 Min**	**120 Min**	**Symptoms**	**Tissue trans-glutaminase**
**1985**	w	neg.	11,2	pos.	4	3	3	2	2	97	99	99	heartburn	neg.	16	15	21	28	18	none	neg.
**1957**	w	neg.	28,1	pos.	5	6	4	5	9	88	98	90	none	neg.	3	3	3	3	5	none	neg.
**1939**	m	neg.	29,3	pos.	1	1	1	1	1	103	112	111	rumbling	neg.	2	1	3	7	5	none	neg.
**1974**	m	neg.	12,3	pos.	3	2	4	9	6	109	113	110	none	neg.	3	2	3	2	2	none	neg.
**1950**	w	neg.	13,6	pos.	2	2	1	6	2	86	81	81	none	neg.	1	1	15	15	4	none	neg.
**1947**	w	neg.	51,6	pos.	3	3	4	8	14	109	119	117	nausea, vertigo	neg.	3	3	4	8	14	none	neg.
**1981**	w	neg.	13,9	pos.	10	10	6	6	5	102	108	89	none	neg.	10	6	6	4	3	none	neg.
**1961**	m	neg.	14,3	pos.	4	6	10	8	15	140	110	129	rumbling, abdominal pain	neg.	4	4	4	4	4	none	neg.
**1952**	m	neg.	13,5	pos.	2	4	2	5	4	124	107	99	none	neg.	1	2	4	3	2	none	neg.
**1980**	w	neg.	16,1	pos.	2	5	7	8	7	100	112	116	none	neg.	20	19	22	24	32	none	neg.
**1979**	m	neg.	12,7	pos.	13	16	12	12	9	118	125	88	none	neg.	10	24	20	13	8	none	neg.
**1964**	m	neg.	12,5	pos.	6	10	7	4	5	107	108	109	none	neg.	13	10	9	8	7	none	neg.
**1993**	m	neg.	17,1	pos.	7	5	4	5	5	134	134	121	diarrhea	neg.	11	8	10	8	7	fullness	neg.
**1967**	w	neg.	15,1	pos.	14	11	8	5	5	110	113	104	none	neg.	14	18	13	10	10	none	neg.
**1982**	w	neg.	17,9	pos.	5	4	3	2	2	107	105	101	none	neg.	4	5	3	2	1	headache	neg.
**1972**	w	neg.	16	pos.	15	11	7	6	8	123	127	104	none	neg.	2	2	3	3	2	none	neg.
**2003**	m	neg.	17,3	pos.	3	5	5	11	11	103	105	97	abdominal pain	neg.	19	12	13	10	10	none	neg.
**1963**	w	neg.	17,7	pos.	5	8	5	2	1	100	103	90	abdominal pain	neg.	3	3	2	3	3	none	neg.
**2002**	m	neg.	16,5	pos.	9	6	3	2	3	96	90	79	headache	neg.	7	13	7	11	5	none	neg.
**1986**	w	neg.	30,6	pos.	17	9	6	4	4	95	93	88	none	neg.	1	12	7	9	6	diarrhea, headache	neg.
**1993**	m	neg.	80	pos.	11	13	9	8	9	114	93	102	abdominal pain	neg.	13	19	15	12	12	none	neg.
**1973**	m	neg.	25,8	pos.	1	2	2	2	2	127	127	127	diarrhea	neg.	8	5	6	6	8	none	neg.
**1993**	w	neg.	16,6	pos.	12	12	6	6	6	88	97	89	none	neg.	5	4	4	4	3	rumbling	neg.
**1978**	m	neg.	37,1	pos.	1	2	1	1	4	110	118	106	abdominal pain	neg.	6	7	4	5	7	abdominal pain	neg.

EXCLI Journal 2022;21:426-435 – ISSN 1611-2156
Received: November 10, 2021, accepted: February 14, 2022, published: February 17, 2022

Year of birth	Gender w (female) m (male)	*H. pylori* (IU/mL)	DAO (IU/mL)	Lactose breath test	Fasting (ppm)	30 Min	60 Min	90 Min	120 Min	Blood glucose fasting	BG 1 (mg%)	BG 2 (mg%)	Symptoms	Fructose breath test	Fasting (ppm)	30 Min	60 Min	90 Min	120 Min	Symptoms	Tissue trans-glutaminase
**1988**	w	neg.	33	pos.	12	15	15	20	15	109	119	118	bloating, vertigo	neg.	15	15	17	19	14	nausea	neg.
**1982**	w	neg.	11	pos.	14	2	0	0	0	107	99	94	abdominal pain, bloating, diarrhea	neg.	5	4	9	1	0	none	neg.
**1969**	m	neg.	25	pos.	2	2	1	2	1	105	113	90	abdominal pain	neg.	3	3	3	2	1	none	neg.
**1981**	m	neg.	12,5	pos.	4	2	0	1	3	95	80	72	none	neg.	3	3	2	2	2	none	neg.
**1997**	w	neg.	11,2	pos.	6	5	6	2	0	84	88	79	nausea	neg.	9	10	6	3	0	none	neg.
**1984**	m	neg.	24,9	pos.	12	10	0	0	4	105	79	89	none	neg.	12	8	2	19	13	none	neg.
**2005**	w	neg.	12,1	pos.	4	8	1	2	2	85	95	87	none	neg.	6	1	1	2	1	none	neg.
**1973**	w	neg.	13,2	pos.	5	5	8	6	7	94	97	87	none	neg.	6	4	3	0	0	none	neg.
**1991**	m	neg.	13,2	pos.	6	30	82	157	151	93	96	96	nausea, vertigo	neg.	13	16	10	10	9	none	neg.
**1985**	m	neg.	12,7	pos.	13	29	47	0	0	83	89	87	tired	neg.	19	20	16	23	20	none	neg.
**1989**	w	neg.	25,8	pos.	14	0	0	0	0	78	91	90	abdominal pain	neg.	10	12	8	3	6	none	neg.
**1999**	m	neg.	13,4	pos.	4	5	7	3	2	102	118	97	none	neg.	1	3	10	0	0	none	neg.
**2001**	w	neg.	13	pos.	5	9	5	3	2	102	89	95	nausea, heartburn	neg.	4	9	12	13	6	none	neg.
**1967**	w	neg.	11,3	pos.	4	9	5	4	4	109	100	94	none	neg.	5	5	9	7	5	none	neg.
**1966**	m	neg.	19,9	pos.	7	13	4	10	3	118	108	119	none	neg.	10	3	22	1	0	none	neg.
**1980**	m	neg.	13,4	pos.	15	14	15	13	11	113	130	110	none	neg.	9	7	23	19	10	rumbling	neg.
**1981**	w	neg.	10,7	pos.	4	2	8	0	0	93	112	99	none	neg.	3	0	0	0	0	none	neg.
**1946**	m	neg.	12,5	pos.	0	0	0	0	0	120	137	112	none	neg.	6	4	0	0	0	none	neg.
**1954**	m	neg.	17,9	pos.	8	5	57	74	76	131	176	170	bloating	neg.	2	8	6	7	2	none	neg.
**1954**	w	neg.	11	pos.	10	11	7	5	4	94	106	111	none	neg.	7	4	5	4	4	none	neg.
**1960**	w	neg.	30,9	pos.	3	4	128	240	298	114	106	113	rumbling	neg.	3	4	6	10	14	none	neg.
**1966**	m	neg.	37,6	pos.	10	3	35	149	118	100	105	106	none	neg.	3	9	7	20	9	none	neg.
**1971**	m	neg.	24	pos.	4	7	5	3	5	114	122	122	none	neg.	3	2	10	10	8	none	neg.

EXCLI Journal 2022;21:426-435 – ISSN 1611-2156
Received: November 10, 2021, accepted: February 14, 2022, published: February 17, 2022

Year of birth	Gender w (female) m (male)	*H. pylori* (IU/mL)	DAO (IU/mL)	Lactose breath test	Fasting (ppm)	30 Min	60 Min	90 Min	120 Min	Blood glucose fasting	BG 1 (mg%)	BG 2 (mg%)	Symptoms	Fructose breath test	Fasting (ppm)	30 Min	60 Min	90 Min	120 Min	Symptoms	Tissue trans-glutaminase
**1960**	m	neg.	21,3	pos.	2	8	27	25	33	99	138	108	none	neg.	0	0	3	0	2	none	neg.
**1983**	w	neg.	15,2	pos.	0	2	22	46	23	114	119	126	bloating	neg.	3	4	1	0	0	none	neg.
**1967**	m	neg.	16,5	pos.	11	10	14	54	35	109	107	90	none	neg.	12	3	3	10	1	none	neg.
**1960**	w	neg.	19	pos.	3	1	1	1	1	115	125	116	none	neg.	1	0	0	0	0	none	neg.

EXCLI Journal 2022;21:426-435 – ISSN 1611-2156
Received: November 10, 2021, accepted: February 14, 2022, published: February 17, 2022

Table 10: Raw data on patients with fructose malabsorption

Year of birth	Gender w (female) m (male)	*H. pylori* (IU/mL)	DAO (IU/mL)	Lactose breath test	Fasting (ppm)	30 Min	60 Min	90 Min	120 Min	Blood glucose fasting	BG 1 (mg%)	BG 2 (mg%)	Symptoms	Fructose breath test	Fasting (ppm)	30 Min	60 Min	90 Min	120 Min	Symptoms	Tissue trans-glutaminase
**1981**	m	neg.	29,9	neg.	9	3	2	4	6	75	98	80	none	pos.	2	10	39	42	32	none	neg.
**1967**	w	neg.	20,8	neg.	20	16	8	10	13	94	108	90	strange taste	pos.	5	12	23	32	37	strange feeling	neg.
**1990**	m	neg.	13,1	neg.	1	1	0	1	1	105	118	103	none	pos.	1	12	25	27	13	none	neg.
**1970**	w	neg.	13	neg.	1	1	1	1	2	74	117	88	none	pos.	14	23	50	103	111	diarrhea, abdominal pain	neg.
**1930**	m	neg.	16,9	neg.	0	1	0	0	0	108	163	134	none	pos.	10	22	36	34	21	none	neg.
**1974**	w	neg.	18,3	neg.	12	11	4	14	24	83	108	95	none	pos.	17	24	62	60	48	none	neg.
**1962**	w	neg.	16,8	neg.	1	4	3	2	4	96	132	94	none	pos.	0	1	16	15	9	bloating, burning tongue	neg.
**1987**	w	neg	19,4	neg.	3	1	0	0	5	86	130	87	none	pos.	0	5	5	28	52	fullness, hunger	neg.
**1966**	m	neg.	18,6	neg.	3	3	2	2	3	118	160	119	none	pos.	4	19	29	26	27	bloating, abdominal pain	neg.
**1961**	m	neg.	10,8	neg.	1	1	2	2	2	109	165	99	none	pos.	0	1	22	10	10	rumbling	neg.
**1943**	m	neg.	13,6	neg.	0	1	0	1	1	97	120	138	none	pos.	0	3	13	38	26	none	neg.
**1977**	w	neg.	62,5	neg.	3	4	4	5	7	92	127	104	none	pos.	1	8	16	21	12	nausea, abdominal pain	neg.
**1948**	m	neg.	20,1	neg.	1	2	5	11	13	135	176	185	none	pos.	3	6	23	22	16	none	neg.
**1965**	m	neg.	11,3	neg.	4	5	3	2	2	102	135	129	none	pos.	14	14	28	33	11	none	neg.
**1985**	m	neg.	25	neg.	2	3	2	2	3	92	100	106	none	pos.	3	7	29	14	18	headache, heartburn	neg
**1977**	m	neg.	20,3	neg.	3	2	2	1	1	108	119	98	bloating	pos.	5	25	18	22	12	rumbling	neg.
**1969**	w	neg.	18,2	neg.	5	6	6	8	6	89	112	75	none	pos.	13	57	37	56	31	rumbling, bloating	neg.
**2002**	m	neg.	12,8	neg.	6	7	5	4	5	84	108	89	rumbling	pos.	1	2	3	27	15	diarrhea, abdominal pain	neg.
**1932**	w	neg.	15,3	neg.	13	14	22	12	8	136	206	153	none	pos.	1	26	32	33	38	none	neg.
**1953**	w	neg.	21,6	neg.	1	1	2	1	0	121	170	120	none	pos.	3	3	7	31	19	none	neg.

EXCLI Journal 2022;21:426-435 – ISSN 1611-2156
Received: November 10, 2021, accepted: February 14, 2022, published: February 17, 2022

Year of birth	Gender w (female) m (male)	*H. pylori* (IU/mL)	DAO (IU/mL)	Lactose breath test	Fasting (ppm)	30 Min	60 Min	90 Min	120 Min	Blood glucose fasting	BG 1 (mg%)	BG 2 (mg%)	Symptoms	Fructose breath test	Fasting (ppm)	30 Min	60 Min	90 Min	120 Min	Symptoms	Tissue trans-glutaminase
**1988**	m	neg.	22,9	neg.	5	4	3	4	9	83	105	64	none	pos.	10	32	50	37	30	none	neg.
**1971**	w	neg.	16,1	neg.	2	3	2	2	2	99	118	92	belching	pos.	4	14	34	40	38	nausea, vertigo	neg.
**1966**	w	neg.	34,1	neg.	1	2	2	4	4	85	97	97	none	pos.	13	17	35	42	46	tiredness, itchy skin	neg.
**1945**	w	neg.	11,1	neg.	2	3	5	13	21	99	164	127	vertigo	pos.	1	17	55	28	20	rumbling, abdominal pain	neg.
**1968**	m	neg.	40,1	neg.	1	1	1	1	2	87	135	103	none	pos.	11	17	33	32	29	tiredness, itchy skin	neg.
**1985**	w	neg.	10,4	neg.	1	2	1	1	1	107	98	122	none	pos.	1	25	38	18	15	rumbling, diarrhea	neg.
**1961**	m	neg.	33,2	neg.	4	4	9	7	8	97	136	113	abdominal pain	pos.	8	11	27	45	36	heartburn	neg.
**1974**	w	neg.	27,3	neg.	4	6	3	7	14	99	162	93	none	pos.	1	8	30	58	40	none	neg.
**1983**	w	neg.	19,1	neg.	6	5	5	3	3	97	107	96	nausea	pos.	1	17	77	30	27	rumbling, bloating	neg.
**1994**	w	neg.	14,5	neg.	3	5	4	3	4	87	107	96	none	pos.	1	4	18	22	11	nausea	neg.
**1948**	w	neg.	17,9	neg.	3	3	4	3	3	98	165	141	rumbling	pos.	1	8	39	26	28	none	neg.
**1985**	w	neg.	10,4	neg.	1	2	1	1	1	107	98	122	none	pos.	1	25	38	18	15	heartburn	neg.
**1977**	w	neg.	14	neg.	2	2	3	13	9	97	112	93	none	pos.	2	2	11	43	74	rumbling	neg.
**1956**	w	neg.	19,2	neg.	1	1	1	1	2	104	142	113	none	pos.	11	6	8	32	8	none	neg.
**1975**	m	neg.	18,5	neg.	1	4	1	1	2	85	153	105	none	pos.	2	40	27	25	8	headache, vertigo	neg.
**1983**	w	neg.	16,8	neg.	2	3	2	2	2	107	130	98	none	pos.	6	11	28	14	12	abdominal pain	neg.
**1994**	m	neg.	12,7	neg.	11	9	8	12	12	108	128	114	nausea	pos.	10	23	51	34	20	rumbling	neg.
**1987**	m	neg.	19,4	neg.	8	11	13	16	24	98	122	101	none	pos.	3	27	21	12	13	abdominal pain	neg.
**1959**	m	neg.	47,1	neg.	6	5	6	6	5	121	154	125	none	pos.	6	29	26	10	11	none	neg.
**1975**	w	neg.	22,1	neg.	5	13	5	4	5	86	137	116	none	pos.	11	18	49	34	19	rumbling	neg.
**1950**	w	neg.	21,4	neg.	1	2	1	0	1	97	121	128	none	pos.	4	9	63	89	104	none	neg.
**1996**	w	neg.	34,1	neg.	11	8	8	8	8	90	135	104	none	pos.	2	3	18	29	24	none	neg.
**1949**	w	neg.	10,2	neg.	16	16	14	12	8	184	273	263	none	pos.	15	37	80	21	14	rumbling	neg.

EXCLI Journal 2022;21:426-435 – ISSN 1611-2156
Received: November 10, 2021, accepted: February 14, 2022, published: February 17, 2022

Year of birth	Gender w (female) m (male)	*H. pylori* (IU/mL)	DAO (IU/mL)	Lactose breath test	Fasting (ppm)	30 Min	60 Min	90 Min	120 Min	Blood glucose fasting	BG 1 (mg%)	BG 2 (mg%)	Symptoms	Fructose breath test	Fasting (ppm)	30 Min	60 Min	90 Min	120 Min	Symptoms	Tissue trans-glutaminase
**1991**	m	neg.	12,5	neg.	11	7	8	6	5	111	130	116	abdominal pain, bloating	pos.	13	16	57	38	26	bloating, rumbling	neg.
**1977**	m	neg.	18	neg.	3	3	2	2	3	107	126	102	none	pos.	8	10	26	33	16	none	neg.
**1952**	w	neg.	26,7	neg.	3	3	5	11	19	120	171	123	nausea, vertigo	pos.	4	15	79	76	62	rumbling	neg.
**1988**	w	neg.	19,8	neg.	17	11	13	9	10	82	99	109	none	pos.	5	3	3	3	39	none	neg.
**1957**	w	neg.	20,1	neg.	12	14	11	10	13	97	151	106	none	pos.	5	9	7	48	146	none	neg.
**1988**	w	neg.	37,1	neg.	13	18	14	13	11	93	159	106	none	pos.	7	18	63	56	46	rumbling, bloating	neg.
**1984**	w	neg.	36,1	neg.	2	3	10	18	21	102	113	100	headache	pos.	2	2	10	21	23	none	neg.
**1956**	w	neg.	15,1	neg.	2	3	3	3	2	103	144	108	none	pos.	1	13	30	26	6	none	neg.
**1967**	w	neg.	16,3	neg.	2	2	2	3	2	101	141	136	none	pos.	4	8	29	18	18	none	neg.
**1956**	w	neg.	15,1	neg.	2	3	3	3	2	103	144	108	none	pos.	1	13	30	26	6	none	neg.
**2001**	m	neg.	16,3	neg.	5	3	5	4	4	78	126	108	none	pos.	9	13	57	22	31	none	neg.
**1985**	w	neg.	15,4	neg.	2	4	4	3	3	84	137	136	none	pos.	10	9	45	36	29	headache, abdominal pain	neg.
**1962**	m	neg.	15	neg.	2	7	11	11	11	105	129	108	none	pos.	2	33	36	33	28	none	neg.
**1993**	m	neg.	20,1	neg.	7	6	6	5	4	84	121	92	none	pos.	16	30	47	68	60	stomach pain	neg.
**1992**	m	neg.	14,4	neg.	12	10	13	7	12	102	135	102	none	pos.	7	11	38	55	64	rumbling	neg.
**1969**	w	neg.	12,2	neg.	8	7	7	8	7	95	125	111	none	pos.	10	31	23	10	15	eczema	neg.
**1987**	m	neg.	18,6	neg.	5	9	7	4	2	93	152	92	none	pos.	7	17	47	25	22	rumbling, fullness	neg.
**1989**	w	neg.	13,6	neg.	7	8	5	7	6	85	175	95	none	pos.	25	54	59	54	26	none	neg.
**1995**	w	neg.	11,7	neg.	12	15	11	9	8	90	111	86	none	pos.	18	26	48	71	58	belching, nausea	neg.
**1977**	w	neg.	12,4	neg.	11	16	19	19	13	94	125	81	none	pos.	18	24	37	46	38	none	neg.
**1941**	w	neg.	19,7	neg.	19	24	21	23	19	155	229	149	none	pos.	21	35	68	98	52	none	neg.
**1956**	w	neg.	15,5	neg.	18	20	15	17	10	94	118	85	none	pos.	0	13	31	40	31	none	neg.
**1982**	w	neg.	15,8	neg.	0	0	0	0	3	84	125	79	none	pos.	4	1	53	49	18	none	neg.
**1996**	w	neg.	18	neg.	0	0	0	0	0	87	129	113	none	pos.	5	0	9	30	23	bloating	neg.

Received: November 10, 2021, accepted: February 14, 2022, published: February 17, 2022

Year of birth	Gender w (female) m (male)	*H. pylori* (IU/mL)	DAO (IU/mL)	Lactose breath test	Fasting (ppm)	30 Min	60 Min	90 Min	120 Min	Blood glucose fasting	BG 1 (mg%)	BG 2 (mg%)	Symptoms	Fructose breath test	Fasting (ppm)	30 Min	60 Min	90 Min	120 Min	Symptoms	Tissue trans-glutaminase
**1952**	w	neg.	14,5	neg.	17	27	19	29	32	103	157	95	none	pos.	5	4	13	21	30	none	neg.
**1982**	m	neg.	11,3	neg.	5	3	6	0	1	103	141	96	none	pos.	8	5	37	33	23	none	neg.
**1952**	w	neg.	18,4	neg.	7	2	0	0	0	105	157	122	none	pos.	3	0	0	0	24	none	neg.
**1952**	m	neg.	14,2	neg.	8	4	0	0	0	101	172	121	vertigo	pos.	0	1	71	10	5	rumbling, diarrhea	neg.
**1989**	m	neg.	12,8	neg.	0	0	0	0	0	109	120	105	none	pos.	8	6	5	16	29	nausea	neg.
**1996**	w	neg.	15,2	neg.	1	2	1	1	2	104	128	80	none	pos.	5	16	17	35	16	none	neg.
**1969**	w	neg.	24,8	neg.	0	1	3	3	9	86	156	119	none	pos.	0	12	27	19	10	abdominal pain	neg.
**1965**	w	neg.	12,9	neg.	1	2	2	4	17	91	145	97	none	pos.	1	3	25	59	68	fullness, headache	neg.
**1969**	w	neg.	24,8	neg.	0	1	3	3	9	86	156	119	none	pos.	0	12	27	19	10	abdominal pain	neg.
**1972**	w	neg.	12,8	neg.	10	18	17	18	14	94	112	101	none	pos.	4	15	74	59	66	abdominal pain	neg.
**1990**	w	neg.	26,3	neg.	0	0	9	8	4	87	125	105	bloating	pos.	0	0	17	27	8	none	neg.
**1979**	w	neg.	23,5	neg.	0	0	7	5	3	103	126	76	heartburn, fullness	pos.	10	31	39	22	14	heartburn, belching	neg.
**1988**	w	neg.	11,5	neg.	0	0	0	0	0	95	130	73	none	pos.	11	45	90	66	37	fullness	neg.
**1984**	m	neg.	14,8	neg.	4	5	4	0	2	99	123	86	none	pos.	8	13	44	50	18	tired	neg.
**1996**	m	neg.	16,6	neg.	0	2	3	1	0	96	122	77	none	pos.	9	25	59	66	102	rumbling	neg.
**1995**	m	neg.	15,4	neg.	11	16	16	11	13	76	133	95	none	pos.	8	199	88	67	38	diarrhea, vertigo, pain	neg.
**1968**	w	neg.	22	neg.	0	0	0	0	0	97	111	95	none	pos.	1	10	17	47	30	rumbling, abdominal pain	neg.
**1960**	w	neg.	34,5	neg.	7	8	8	4	4	99	151	85	none	pos.	0	3	4	4	24	tired	neg.
**1958**	m	neg.	12,4	neg.	0	0	0	0	0	101	152	143	none	pos.	1	32	43	18	10	abdominal pain, nausea	neg.
**1995**	m	neg.	13,9	neg.	9	2	3	1	0	96	112	83	none	pos.	3	27	60	60	29	diarrhea	neg.
**1944**	w	neg.	25	neg.	0	0	0	0	0	114	145	122	none	pos.	0	0	44	66	16	none	neg.
**1978**	w	neg.	20,6	neg.	1	3	2	3	7	86	138	101	headache	pos.	6	39	44	20	16	none	neg.
**1943**	m	neg.	16,2	neg.	1	4	3	2	6	118	165	113	none	pos.	5	7	32	46	27	none	neg.

EXCLI Journal 2022;21:426-435 – ISSN 1611-2156
Received: November 10, 2021, accepted: February 14, 2022, published: February 17, 2022

**Year of birth**	**Gender w (female) m (male)**	***H. pylori* (IU/mL)**	**DAO (IU/mL)**	**Lactose breath test**	**Fasting (ppm)**	**30 Min**	**60 Min**	**90 Min**	**120 Min**	**Blood glucose fasting**	**BG 1 (mg%)**	**BG 2 (mg%)**	**Symptoms**	**Fructose breath test**	**Fasting (ppm)**	**30 Min**	**60 Min**	**90 Min**	**120 Min**	**Symptoms**	**Tissue trans-glutaminase**
**2001**	w	neg.	12,4	neg.	5	2	0	3	8	90	134	68	none	pos.	4	11	57	57	36	abdominal pain, bloating	neg.
**1994**	w	neg.	10,6	neg.	7	4	6	10	3	83	127	83	none	pos.	1	9	23	9	0	none	neg.
**1945**	w	neg.	12,8	neg.	0	1	3	3	9	98	153	122	none	pos.	1	3	57	106	103	none	neg.
**2001**	w	neg.	22,6	neg.	12	13	11	3	1	90	133	92	none	pos.	11	5	16	42	20	none	neg.
**1956**	w	neg.	15,5	neg.	2	2	3	2	4	171	193	163	none	pos.	0	1	13	31	45	diarrhea	neg.
**1993**	w	neg.	18,7	neg.	16	29	21	17	17	77	112	95	nausea	pos.	17	35	62	64	60	none	neg.
**1989**	m	neg.	10,9	neg.	2	6	6	1	9	101	148	89	none	pos.	4	41	22	11	7	dyspnea	neg.
**2000**	w	neg.	15,6	neg.	2	6	17	6	1	111	140	107	none	pos.	9	4	32	19	13	none	neg.
**1982**	w	neg.	29,3	neg.	10	25	19	16	4	131	188	120	none	pos.	12	49	43	21	28	bloating	neg.
**1950**	w	neg.	16,2	neg.	1	7	12	4	6	102	136	106	none	pos.	0	25	29	19	13	none	neg.
**1969**	m	neg.	25,2	neg.	4	6	15	14	16	117	145	117	none	pos.	11	8	28	44	24	none	neg.
**1961**	w	neg.	13,4	neg.	9	14	16	11	13	137	169	106	none	pos.	1	6	69	72	21	diarrhea	neg.
